# Linking lungs and gums: a meta-analysis of periodontitis prevalence and severity in chronic obstructive pulmonary disease

**DOI:** 10.1038/s41405-026-00403-6

**Published:** 2026-02-09

**Authors:** Gowri Sivaramakrishnan, Kannan Sridharan

**Affiliations:** 1 Bahrain Defence Force Royal Medical Services, Riffa, Bahrain; 2 Department of Pharmacology and Therapeutics, College of Medicine and Health Sciences, Arabian Gulf University, Manama, Bahrain

**Keywords:** Periodontitis, Periodontitis

## Abstract

**Aim:**

Chronic Obstructive Pulmonary Disease (COPD) is a progressive respiratory disorder associated with chronic inflammation and airflow limitation. Periodontitis shares common risk factors with COPD, such as smoking. This meta-analysis evaluates the prevalence and severity of periodontitis in COPD patients by assessing periodontal parameters, including probing depth (PD), clinical attachment loss (CAL), and bleeding on probing (BOP).

**Methods:**

A systematic search of electronic databases identified clinical studies reporting periodontitis in COPD patients. Studies with clear diagnostic criteria for both conditions were included. Data extraction was conducted using RAYYAN software, and study quality was assessed using the Newcastle-Ottawa Scale (NOS). A random-effects meta-analysis was performed in R Studio, with heterogeneity assessed using the I² statistic. To assess the robustness of the findings, a leave-one-out sensitivity analysis was conducted for CAL and PD. Publication bias was examined using the Trim and Fill method. Additionally, stratified subgroup analyses were performed based on smoking status and study design to explore potential sources of heterogeneity. Certainty of evidence was assessed using GRADE.

**Results:**

A total of 41 studies were included. Periodontitis prevalence in COPD patients was 35%, significantly higher than in non-COPD controls. COPD patients exhibited greater periodontal destruction, with increased CAL (0.68 [0.37;0.98]) deeper PD (0.72 [0.12; 1.31]), and BOP (1.49 [0.19;2.79]). However, the certainty of this evidence was rated as low to very low.

**Conclusion:**

COPD patients have a higher prevalence of periodontitis and worse periodontal parameters. While our meta-analysis suggests an association, the overall certainty of evidence is low. These findings should therefore be interpreted with caution. Future high-quality prospective studies are essential to confirm this relationship and inform clinical practice.

## Introduction

Chronic Obstructive Pulmonary Disease (COPD) is a prevalent and progressive condition characterized by persistent airflow limitation, which manifests as chronic cough, sputum production, and dyspnea [1]. According to a recent data, the overall global prevalence of COPD is 12.64% using the fixed ratio (FR) definition and 7.38% using the lower limit of normal (LLN) criteria [1]. The prevalence is higher in men (15.47%) compared to women (8.79%). The highest prevalence was found in the Americas using the FR definition and in Southeast Asia using the LLN definition. The most common COPD stage was stage II, with a prevalence of 50.46%. The findings also highlighted significant regional data gaps, particularly in Africa and the Eastern Mediterranean [1]. Key risk factors for COPD included male sex, smoking (current and ever), low body mass index (BMI < 18.5 kg/m²), biomass exposure, and occupational exposure to dust or smoke [2].

Periodontitis, a prevalent chronic inflammatory disease affecting the supporting structures of teeth, has been shown to have a significant association with various systemic diseases, including COPD. A recent meta-analysis [3] aimed to assess the global prevalence of periodontitis between 2011 and 2020. The prevalence of periodontitis in dentate adults was ~62%, with severe periodontitis affecting 23.6% of the population [3]. Studies using confident case definitions such as the American Association of Periodontology/Center for Disease Control (AAP/CDC) 2012 reported a pooled periodontitis prevalence of 61.6%, nearly twice the rate of those with non-confident definitions such as community periodontal index (CPI) of 3 or 4, periodontal pocket depth >4 mm, and clinical attachment level ≥1 mm, which was 38.5%. Among the confident case definitions, the AAP/CDC 2012 provided the highest estimate at 68.1%, while the AAP/CDC 2007 presented the lowest at 48.8%. Age was identified as a major confounding variable, with older participants (≥65 years) showing the highest pooled estimate of 79.3% [3].

Both periodontitis and COPD are highly prevalent conditions that significantly impact the quality of life of individuals [4]. They share several common risk factors, such as smoking, microbial infections, environmental pollution, diabetes, poor socio-economic status, and inadequate dental care practices [5]. It is increasingly recognized that periodontal pathogens, particularly *Porphyromonas gingivalis, Fusobacterium nucleatum, Treponema denticola*, and *Aggregatibacter actinomycetemcomitans*, can contribute to systemic inflammation and have detrimental effects beyond the oral cavity [6]. Additionally, periodontal disease may promote the colonization of respiratory pathogens in the mouth, which are known to exacerbate COPD [7, 8].

In COPD patients, the elevated expression of mucin proteins, particularly Mucin 5AC (MUC5AC), induced by periodontal pathogens such as *Porphyromonas gingivalis and Fusobacterium nucleatum*, may contribute to mucous hypersecretion and airway obstruction, further compromising lung function [9]. Moreover, the transfer of pathogens from the oral cavity to the lungs can provoke an inflammatory response in the respiratory epithelium, further impairing lung function. *P. gingivalis* has been shown to activate the toll-like receptor-2 (TLR-2) and NF-κB signaling pathways, leading to the production of pro-inflammatory cytokines such as TNF-α, IL-17, and G-CSF, worsening COPD symptoms [8].

Previous meta-analyses [10–19] examining the link between COPD and periodontitis, pooled data from various respiratory diseases, included fewer studies, did not analyze specific periodontal parameters individually, or failed to report according to standard reporting guidelines (Table 1). Additionally, they overlooked studies focused on prevalence. Hence, the aim of this meta-analysis is to specifically target COPD to evaluate the impact of key periodontal health indicators—such as probing/pocket depth (PD), clinical attachment loss (CAL), and bleeding on probing (BOP)—in COPD patients, comparing them with participants without COPD. The study aims to assess the prevalence and severity of periodontal disease in individuals with COPD, providing insights into the potential links between the two conditions.

**Table 1:** Systematic reviews and meta-analyses on COPD and periodontitis.

Study id	Results and conclusions
Apessos 2021	A review of 7 full-text articles, out of 1442 retrieved, found limited evidence suggesting that periodontal treatment in patients with COPD and periodontitis is associated with reduced exacerbation frequency and a slower decline in lung function. However, the effects on quality of life remained unclear. Additionally, periodontal treatment was linked to lower hospitalization rates and reduced all-cause mortality in COPD patients. Despite these findings, significant methodological differences among the studies were noted, and the overall quality of evidence was deemed very low to moderate.
Azarpazhooh 2006	A review of 19 studies identified key findings related to oral health and respiratory diseases. The studies revealed that the presence of cariogenic and periodontal pathogens, dental decay, and poor oral hygiene were significant risk factors for pneumonia. A weak association was found between periodontal disease and COPD in four studies, with an odds ratio (OR) of less than 2. Ten studies provided evidence that improving oral health through oral hygiene interventions reduced the progression or occurrence of pneumonia. The review concluded that there is fair evidence of an association between poor oral health and pneumonia (OR = 1.2–9.6), poor evidence of a weak link between periodontal disease and COPD (OR < 2.0), and strong evidence that improved oral hygiene and regular professional care reduce the progression or occurrence of respiratory diseases, particularly in high-risk elderly adults and those in intensive care units.
Gomes-Filho 2019	A systematic review of 13 studies, with 10 contributing to meta-analysis, found that periodontitis was significantly associated with asthma (adjusted OR: 3.54, 95% CI: 2.47–5.07), COPD (adjusted OR: 1.78, 95% CI: 1.04–3.05), and pneumonia (adjusted OR: 3.21, 95% CI: 1.99–5.17). The heterogeneity among studies was low for asthma and pneumonia (I^2^ = 0%), and moderate for COPD (I^2^ = 37.9%). The review concluded that periodontitis is validated as an associated factor for asthma, COPD, and pneumonia.
Kelly 2021	8 studies met the inclusion criteria, including three clinical trials, one prospective cohort study, one case–control study, and three cross-sectional studies. A narrative synthesis was performed, showing that intervention studies indicated a reduction in the frequency of COPD exacerbations following periodontal treatment. Observational studies suggested that worse plaque scores and fewer teeth were associated with exacerbations, but pocket depth or clinical attachment loss were not. Better periodontal health was linked to reduced exacerbation frequency, fewer hospitalizations, and improved quality of life in COPD patients. Due to high heterogeneity, no meta-analysis was conducted. The quality of some studies was low, with a high risk of bias. The data supports a possible association between poor periodontal health and exacerbations, hospitalizations, and quality of life in COPD patients. However, the evidence is of moderate to low certainty, highlighting the need for well-designed, adequately powered randomized controlled trials to inform future research and clinical practice.
Molina 2022	Meta-analyses of 75 articles revealed significant associations between periodontitis and COPD (OR = 1.28, 95% CI [1.16; 1.42], *p* < 0.001) and periodontitis and OSA (OR = 1.65, 95% CI [1.21; 2.25], *p* = 0.001). However, no significant association was found for asthma (OR = 1.53, 95% CI [0.82; 2.86], *p* = 0.181). For acute respiratory conditions, two studies reported results for CAP, while significant associations were found for COVID-19 complications, including the need for assisted ventilation (OR = 6.24, 95% CI [2.78; 13.99], *p* < 0.001) and COVID-related mortality (OR = 2.26, 95% CI [1.36, 3.77], *p* = 0.002). Despite these associations, only four intervention studies were identified, showing positive effects of periodontal treatment on COPD, asthma, and CAP. The review concludes that periodontitis is positively associated with COPD, OSA, and complications from COVID-19, but there is a lack of intervention studies to assess the clinical benefits of periodontal treatment.
Scannapieco 2003	36 met the inclusion criteria, with 21 studies (11 case-control and cohort studies with 1413 participants and 9 RCTs with 1759 participants) included in the final analysis. Key findings include: 1) oral interventions that improve hygiene through mechanical or chemical disinfection, or antibiotics, reduced the incidence of nosocomial pneumonia by ~40%; 2) several studies showed a potential link between periodontal disease and COPD. The review concludes that oral colonization by respiratory pathogens, due to poor oral hygiene and periodontal disease, appears to be associated with nosocomial pneumonia. However, further large-scale RCTs are needed to establish effective oral hygiene procedures to prevent nosocomial pneumonia in high-risk patients. The association between periodontal disease and COPD remains preliminary, with the need for larger longitudinal, epidemiological, and RCT studies to confirm the relationship.
Shi 2018	This meta-analysis included 14 studies with 3348 COPD patients and 20,612 non-COPD controls, analyzing 9 periodontal indices. The results showed that COPD patients had significantly worse periodontal health compared to non-COPD subjects, with higher probing depth (mean difference 0.261), greater clinical attachment loss (0.480), and more alveolar bone loss (0.127). Additionally, COPD patients had worse oral hygiene (plaque index 0.802), increased bleeding on probing (6.878), and a lower number of remaining teeth (−3.726). The findings indicate that COPD patients suffer from deeper periodontal pockets, higher levels of clinical attachment loss, more gingival inflammation and bleeding, and poorer oral hygiene. However, the authors note that more high-quality, well-designed studies are needed to confirm these results and further investigate the periodontal health of COPD patients.
Wu 2022	This meta-analysis included 37 studies and revealed a significant association between pulmonary and periodontal disease (adjusted OR: 1.93; 95% CI: 1.60–2.33; *P* < 0.05). The pooled adjusted ORs for COPD, asthma, and pneumonia were 1.64, 3.03, and 2.21, respectively. The analysis also showed that patients with pulmonary diseases had worse periodontal health, as most periodontal indices in these patients were poorer. The review concludes that there is a strong association between pulmonary disease and periodontal health, highlighting the need for clinical trials to investigate the causality and underlying pathological mechanisms of this association.
Yang 2023	This meta-analysis included 22 observational studies with 51,704 participants. The pooled analysis of 18 studies showed a weak association between periodontal disease (PD) and the risk of COPD (OR: 1.20, 95% CI 1.09–1.32). However, when the analysis was adjusted for smoking intensity, the association was no longer significant (OR: 1.14, 95% CI 0.86–1.51). Further stratified analyses revealed no significant relationship between periodontal disease and COPD risk in smokers (OR: 1.46, 95% CI 0.92–2.31) or never-smokers (OR: 0.93, 95% CI 0.72–1.21). Additionally, Periodontitis did not increase the risk of COPD-related exacerbations or mortality (OR: 1.18, 95% CI 0.71–1.97). The study concludes that PD does not confer a risk for COPD or related events when strictly adjusted for smoking. It suggests that large-scale prospective cohort studies are needed to validate these findings.
Zeng 2012	This meta-analysis included 14 observational studies (one nested case-control, eight case-control, and five cross-sectional) with 3988 COPD patients. The results indicated a significant association between periodontal disease and COPD, with an odds ratio of 2.08 (95% CI = 1.48–2.91, *p* < 0.001). Sensitivity analysis confirmed the robustness of the result. Subgroup analyses, including study design, ethnicity, assessment of periodontal disease/COPD, and adjusted/unadjusted odds ratios, also revealed a significant association. Publication bias was detected. The study concludes that PD is a significant and independent risk factor for COPD, although the causal relationship remains unclear. The authors recommend further randomized controlled trials to explore whether periodontal interventions can influence COPD pathogenesis and progression.

*CI* Confidence interval, *COPD* Chronic obstructive pulmonary disease, *OR* Odds ratio, *OSA* obstructive sleep apnea, *CAP* Community acquired pneumonia.

## Methods

### Protocol and registration

The protocol for this meta-analysis was registered in PROSPERO with the registration number CRD42024625280. The protocol can be assessed at https://www.crd.york.ac.uk/prospero/#recordDetails.We complied with the reporting guidelines outlined in the PRISMA (Preferred reporting items for systematic reviews and Meta-analysis) [20]. Considering the nature of this study, ethics approval was not sought.

### Data sources and search strategy

The following electronic databases were searched until January 15, 2025: PUBMED, Scopus, Embase, and Web of Science. References from eligible articles were also considered, wherever appropriate, when unavailable from the above databases through the framed search strategy. The search strategy used in different databases is provided in Supplementary File 1. For improving the sensitivity of the electronic search, the keywords were used alone and in combination.

### Study selection and eligibility criteria

We imported the articles that were identified following the search to RAYYAN systematic review software [21]. The duplicates were removed thereafter, and two independent reviewers (GS and KS) screened titles and abstracts of all articles for their suitability. Full texts were obtained for the potential articles for inclusion for determining if they met the specified inclusion criteria. Any discrepancies between the reviewers were resolved through discussion for reaching a consensus.

### Inclusion and exclusion criteria

All clinical studies conducted globally on patients with COPD that reported the outcomes of interest were included. The included studies may or may not have a control group. Clear diagnostic criteria for COPD and periodontitis must be indicated for inclusion. The study designs included were observational studies, case control, cohort studies, cross-sectional studies, randomized and non-randomized clinical studies. Only peer-reviewed studies published in indexed journals were eligible for inclusion.

The primary outcome of interest was the number of participants with periodontitis in both the case and the control group. Subgroup analysis was conducted for severity of periodontitis. Stratified analysis were carried out based on the study types and smoking status (less than 50% and greater than 50%) of the study participants in COPD group.

Other outcomes that were measured were: number of patients with PD and CAL > 3 mm, number of patients with >5 mm PD, mean BOP scores, mean CAL scores and mean PD scores. No restriction was placed on the year of publication. Case reports, case series, opinion papers, letters, review articles, and those studies that did not report the necessary data on the outcome of interest were excluded.

The PICO is presented below:

Population (P): Participants with or without COPD for whom periodontal parameters such as prevalence of periodontitis, PD, BOP and CAL were measured.

Intervention group (E): Patients with COPD.

Comparator (C): Patients without COPD or no control group

Outcome (O): Prevalence of periodontitis, mean BOP scores, mean PD, mean CAL.

### Data extraction and study quality assessment

Data related to study design, geographic location, age, year of publication, sample size, were collected. The included studies were assessed for quality using the Newcastle-Ottawa Scale (NOS) for non-randomized studies (Supplementary File 2). Each study was evaluated using a star system, where each question could receive either one or two stars based on the quality of the reporting. Studies were subsequently categorized as good, satisfactory, or unsatisfactory, depending on the total number of stars received. This systematic evaluation of study quality helped to ensure the reliability of the findings and conclusions drawn from the analysis.

### Statistical analyses

This meta-analysis was conducted using R Studio software with the meta, metafor, and metaprop packages. Random-effects meta-analysis was carried out for estimating the effect sizes between the interventions. The effect sizes for the outcomes were represented either as odds ratios (OR) or standardized mean difference (SMD) and 95% confidence intervals (CI). We used the SMD instead of the mean difference (MD) to account for variations in measurement scales across studies, ensuring comparability and facilitating the synthesis of effect sizes in a random-effects model. In addition to effect size estimation, we performed an analysis of proportion to determine the prevalence of periodontitis in the included studies.

Heterogeneity among the included studies was assessed using the I² statistic. I² values were interpreted as follows: 0–25% representing low heterogeneity, 26–50% as moderate heterogeneity, 51–75% as substantial heterogeneity, and 76–100% as considerable heterogeneity. Forest plots were generated to visually represent the effect sizes and their corresponding 95% CI. The diamond at the bottom of the plot indicates the overall pooled effect size.

To assess potential publication bias, we applied the trim-and-fill method, which estimated the number of missing studies and adjusted the pooled effect size accordingly. The adjusted effect size was then recalculated, providing a more accurate estimate of the true effect. If the adjusted effect size remains similar to the original estimate, publication bias is unlikely to have a significant impact on the results. Additionally, a leave-one-out sensitivity analysis was conducted for key outcomes to evaluate the influence of individual studies on the overall pooled estimate. This sensitivity analysis involved systematically removing one study at a time and recalculating the pooled effect size, helping to identify any studies that might disproportionately affect the results. The certainty of evidence for primary outcomes was assessed using the Grading of Recommendations Assessment, Development and Evaluation (GRADE) approach.

## Results

### Study results

**Fig. 1:**
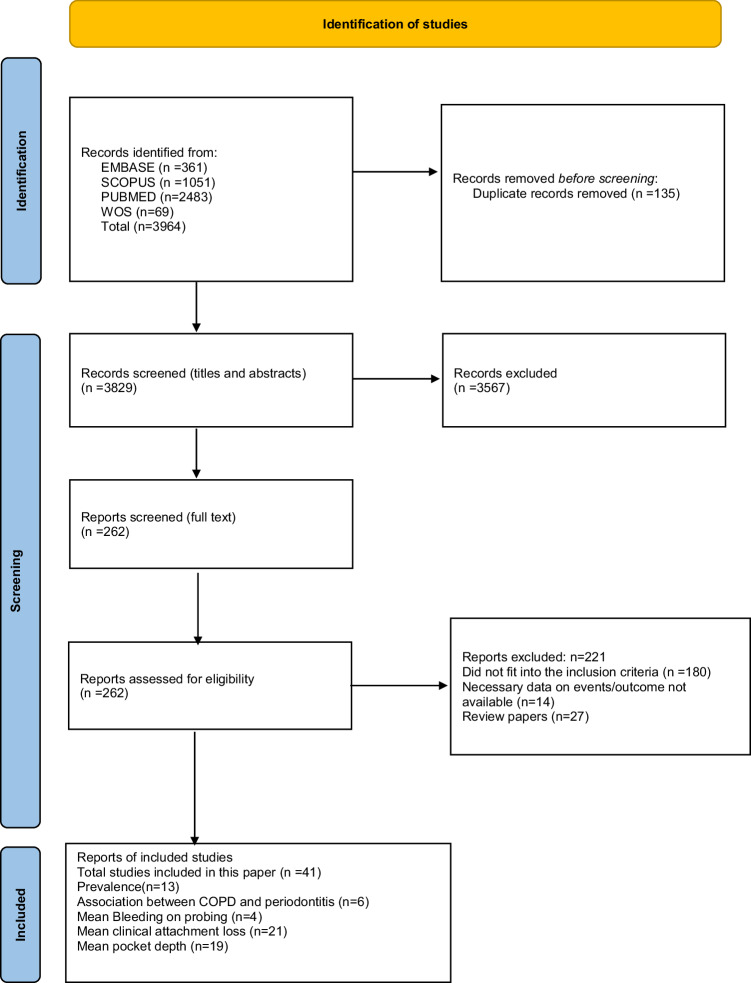
PRISMA flow diagram.

The PRISMA flow diagram (Fig. 1) outlines the study selection process for this meta-analysis.

Out of 3964 identified records, 135 duplicates were removed, leaving 3829 studies for screening. After title and abstract screening, 262 full-text articles were assessed. Ultimately, 41 studies [22–62] were included in the final analysis, ensuring a transparent and systematic selection process. The key characteristics of included studies are presented in Table 2. All included studies were observational, consisting mainly of cross-sectional, case-control, and cohort designs; no randomized controlled trials were identified.

**Table 2:** Key characteristics of included studies.

Study Id	Country	Study design	Case group	N1	Age mean (SD)/mean (range)/range	Control group	N2	Age mean (SD)	NOS categories
Baldomero 2019	USA	Case control study	COPD	136	67.1 (6.5)	No control	NA	NA	Satisfactory
Barros 2013	USA	Cohort	COPD	1635	65.4 (5.3)	No control	NA	NA	Satisfactory
Bergstrom 2013	Sweden	Cohort	COPD	52	54 (38–72)	Without COPD	28	61 (48–78)	Good
Bomble 2020	India	Cohort	COPD	39	35-75	Without COPD	78	Age matched	Good
Chrysanthakopoulos 2014	Greece	Cross-sectional	COPD	302	54.7 (4.4)	No control	NA	NA	Satisfactory
Chrysanthakopoulos 2020	Greece	Case control study	COPD	392	Greater than 40	Without COPD	1803	Greater than 40	Good
Chung 2016	Korea	KNHANES study	COPD	697	64.3 (0.2)	Without COPD	5181	54.6 (0.1)	Good
Lopez-de-Andrés 2018	Spain	National European survey	COPD	2721	Greater than 40	Without COPD	2721	Greater than 40	Good
Deo 2009	India	Case control study	COPD	150	41.43 (7.53)	Without COPD	50	43.62 (5.53)	Good
Gupta 2020	India	Case control study	COPD	65	50.31 (11.4)	Without COPD	65	41.91 (41.91)	Satisfactory
Harland 2018	Japan	Cross-sectional	COPD	149	61.3 (9.1)	Without COPD	1325	54.5 (8.7)	Good
Henke 2016	Germany	Cross-sectional	COPD	206	42.0 (32.0; 54.7)	No control	NA	NA	Satisfactory
Hyman 2004	USA	NHANES study	COPD	993	62.32 (14.07)	Without COPD	6632	47.37 (14.23)	Good
Javaheri 2020	Iran	Case control study	COPD	36	57.6 (11.04)	Without COPD	36	56.58 (10.83)	Good
Jung 2020	Korea	KNHANES study	COPD	1134	54.71 (0.17)	Without COPD	6585	Age matched	Good
Katancik 2005	USA	Prospective cohort	COPD	75	74.6 (2.8)	Without COPD	785	Age matched	Poor
Kedlaya 2021	India	Cross-sectional	COPD	20	59.5 (4.7)	Without COPD	40	Age matched	Good
Komerik 2005	Turkey	Cross-sectional	COPD	30	65.9 (11.0)	Without COPD	30	66.2 (8.4)	Satisfactory
Kucukcoskun 2013	Turkey	Cross-sectional	COPD	40	59.83 (10.09)	No control	NA	NA	Satisfactory
Ledic 2013	Croatia	Cross-sectional	COPD	93	65.75 (9.65)	Without COPD	43	62.12 (11.91)	Good
Liu 2012	China	Cross-sectional	COPD	392	63.9 (9.8)	No control	NA	NA	Satisfactory
Liu 2023	China	Cross-sectional	COPD	4759	60.36 (11.39)	No control	NA	NA	Satisfactory
Offenbacher 2012	USA	Cross-sectional	COPD	2232	64.9 (5.57)	Without COPD	8422	62.4 (5.59)	Good
Öztekin 2014	Turkey	Cross-sectional	COPD	52	57.5 (9.7)	Without COPD	38	53.5 (9.2)	Good
Peter 2013	India	Observational	COPD	102	59.48 (11.13)	Without COPD	399	49.69 (10.16)	Good
Prasanna 2011	India	Observational	COPD	50	56.38 (3.89)	Without COPD	50	47.40 (4.98)	Good
Raj 2018	India	Observational	COPD	170	36.80 (7.10)	Without COPD	170	35.83 (7.36)	Good
Sanghavi 2024	India	Observational	COPD	50	Above 18 years	Without COPD	50	Age matched	Good
Sapey 2019	UK	Cohort	COPD	156	62.9 (31–82)	No control			Satisfactory
Sathyanarayana 2023	India	Case control study	COPD	285	49.89 (9.4)	Without COPD	285	50.06 (9.03)	Good
Scannapieco 2001	USA	NHANES study	COPD	810	51.2 (17.9)	Without COPD	12982	43.9 (17.7)	Good
Sepolia 2021	India	Cross-sectional	COPD	150	20-60	Without COPD	150	Age matched	Good
Si 2012	China	Cross-sectional	COPD	581	63.9 (9.4)	Without COPD	438	62.8 (9.5)	Satisfactory
Subappa 2023	India	Cross-sectional	COPD	199	64.5 (8.8)	No control	NA	NA	Satisfactory
Takeuchi 2019	Japan	Prospective cohort	COPD	900	68.8 (6.3)	No control	NA	NA	Satisfactory
Tan 2019	China	Case control study	COPD	80	60.7 (11.9)	Without COPD	80	61.0 (9.0)	Good
Tereshima 2016	Japan	Case control study	COPD	60	73.9 (7.6)	Without COPD	76	65.1 (5.8)	Good
Wang 2009	china	Case control study	COPD	306	63.94 (9.84)	Without COPD	328	63.26 (8.98)	Good
Winning 2019	Sweden	Cross-sectional	COPD	86	60-96	Without COPD	740	60-96	Satisfactory
Yildirim 2013	Turkey	Cross-sectional	COPD	36	68.9 (8.8)	Without COPD	20	59.5 (9.1)	Good
zhou 2020	China	Case control study	COPD	60	63.1 (10.1)	Without COPD	60	60.0 (9.4)	Good

*COPD* chronic obstructive pulmonary disease, *SD* Standard deviation, *NOS* Newcastle-Ottawa scale.

Most of the included studies diagnosed COPD using spirometry, with the case definition based on a FEV1/FVC ratio of less than 0.70, following the guidelines set by the American Thoracic Society/European Respiratory Society [2, 63]. Other diagnostic methods involved physician diagnosis, typically associated with indications for lung transplantation, chronic bronchitis, or emphysema, as well as a combination of pulmonary function tests, chest radiographs, and physical examinations. The diagnostic criteria for periodontitis reported in these studies is presented in Table 3. The Risk of bias using NOS categories are also presented in Table 2. The NOS scores of the included studies were generally categorized as Good (5–7 points), with most studies falling within this range, indicating moderate to high methodological quality.

**Table 3:** Diagnostic criteria of periodontitis in the included studies on prevalence.

Study id	Description of periodontitis severity classification based on
Baldomero 2009	American Academy of Periodontology Task Force Report
Ciardo 2023	Consensus report of workgroup 2 of the 2017 World Workshop on the Classification of Periodontal and Peri-Implant Diseases and Conditions.
Offenbacher 2012	American Association of Periodontology/Center for Disease Control (AAP/CDC) consensus definitions of Health, Early and Advanced disease
Sapey 2019	American Association of Periodontology/Center for Disease Control (AAP/CDC) consensus definitions of Health, Early and Advanced disease
Si 2012	Moderate periodontitis = ≥1 teeth with ≥5mmPD or 30% to 60% of the teeth examined having ≥4 mm PD severe periodontitis = ≥30% of the teeth examined having ≥5 mm PD or ≥60% of the teeth examined having ≥4 mm PD.
Subappa 2023	2018 World Workshop on the Classification of Periodontal and Peri-Implant Diseasesand Conditions
Takeuchi 2019	American Association of Periodontology/Center for Disease Control (AAP/CDC) consensus definitions of Health, Early and Advanced disease
Barros 2013	American Association of Periodontology/Center for Disease Control (AAP/CDC) consensus definitions of Health, Early and Advanced disease
Liu 2023	Severe periodontitis questionnaire - The specific questions that interviewers asked were “Have you had loose teeth in the past year?”, and “Have you had spontaneous tooth loss in the past year?”. The answers for these two questions were both set as “Yes” or “No”. Participants who answered “Yes” for at least one of the two questions were defined as having symptoms of severe periodontitis.
Chung 2016	Periodontitis was defined as a CPI 3 (>3.5-mm to ≤5.5-mm pocket) or CPI 4 (>5.5 mm pocket).
Bergstrom 2013	A mean pocket depth of ≥4 mm was arbitrarily chosen as cut off point to define periodontal disease.
Lopez de-Andres 2018	Questionnaire - Participants were considered as periodontal disease sufferers if when asked “Do you suffer of any of these dental and oral disorders or disease?” they answered “my teeth bleed spontaneously or while brushing” or/and “my teeth move.”
Henke 2016	The presence of periodontitis was defined according the following criteria:Periodontal screening index ≥3 or previous treatment of periodontitis, or bone loss ≥1.
Harland 2018	Periodontitis was defined as the presence of at least one sextant with a pocket depth ≥4 mm (CPI score ≥3)
Leuckfeld 2008	Periodontitis was defined as a mean marginal bone level ≥4 mm

*PD* pocket depth, *CPI* Community periodontal index.

### Prevalence of periodontitis in COPD patients

**Fig. 2:**
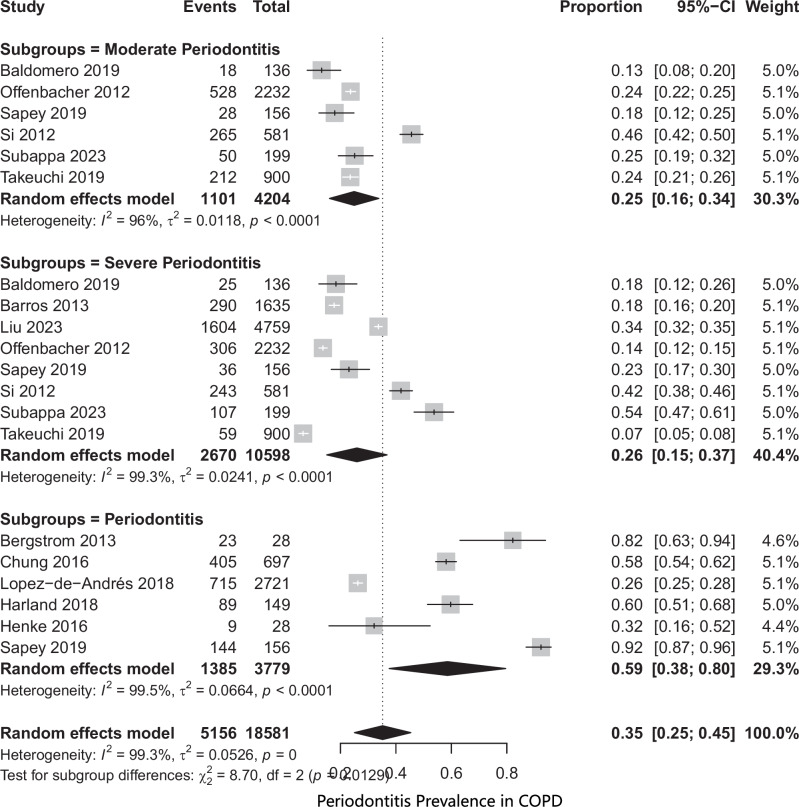
Periodontitis prevalence.

Thirteen studies [29, 31, 32, 34, 37, 41, 42, 44, 46, 48, 49, 55, 61] reported the prevalence of periodontitis in 18581 patients. The pooled prevalence was 35%. Subgroup analysis showed that there was a 25% prevalence of moderate periodontitis and 26% prevalence of severe periodontitis in COPD patients (Fig. 2).

Stratified analysis of the COPD group based on smoking status revealed that studies with more than 50% smokers reported a pooled periodontitis prevalence of 44%, including 34% with severe periodontitis and 30% with moderate periodontitis (Supplementary File 3). In contrast, studies with less than 50% smokers in COPD group showed a significantly lower pooled prevalence of 19%, comprising 21% moderate and 18% severe periodontitis (Supplementary File 4). The pooled prevalence of periodontitis in COPD patients was 37% in cohort studies and 38% in cross-sectional studies (Supplementary File 5). Leave-one-out sensitivity analysis demonstrated that the pooled prevalence ranged from 33% to 37% when each study was omitted one at a time (Supplementary File 6).

A Trim-and-Fill analysis was conducted to assess potential publication bias for this outcome. (Supplementary File 7) The Trim-and-Fill method imputed six studies, and after adjustment, the recalculated pooled effect size was 0.22 [0.09; 0.34]. The result remained statistically significant, suggesting that the estimate is robust to potential small-study effects.

### Association between Periodontitis and COPD

**Fig. 3:**
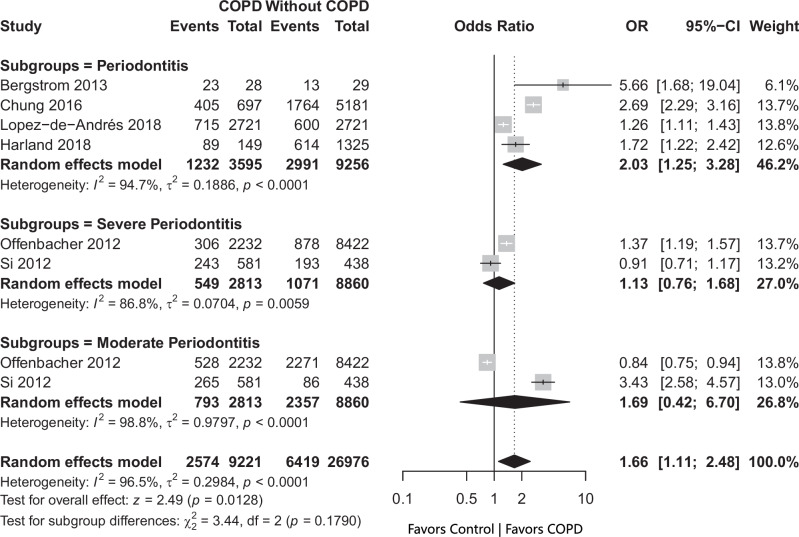
Periodontitis OR.

Six studies [29, 32, 34, 41, 44, 46] reported the prevalence of Periodontitis in COPD patients compared to controls without COPD (Fig. 3). The pooled estimates showed that there was a significantly greater odds of periodontitis in patients with COPD. The odds ratio was 1.66 [1.11; 2.48]. Leave-one-out sensitivity analysis further confirmed this association, showing consistently elevated odds ranging from 1.4 to 1.7 when each study was omitted one at a time (Supplementary File 8).

The Trim-and-Fill method imputed three studies, and after adjustment, the recalculated pooled effect size was 1.13 [0.66; 1.91]. The 95% confidence interval crosses the null value, indicating that the statistical significance of this association is sensitive to funnel plot asymmetry and potential small-study effects, including possible publication bias (Supplementary File 9).

### Mean BOP scores

Four studies [35, 36, 40, 57] reported mean BOP in patients with COPD. All studies had a control group without COPD except Kucukcoskun et al. [36]. The pooled SMD of 1.49 [0.19, 2.79], indicates a significantly higher mean BOP scores among COPD patients compared to non-COPD individuals. (Supplementary File 10).

### Mean CAL

**Fig. 4:**
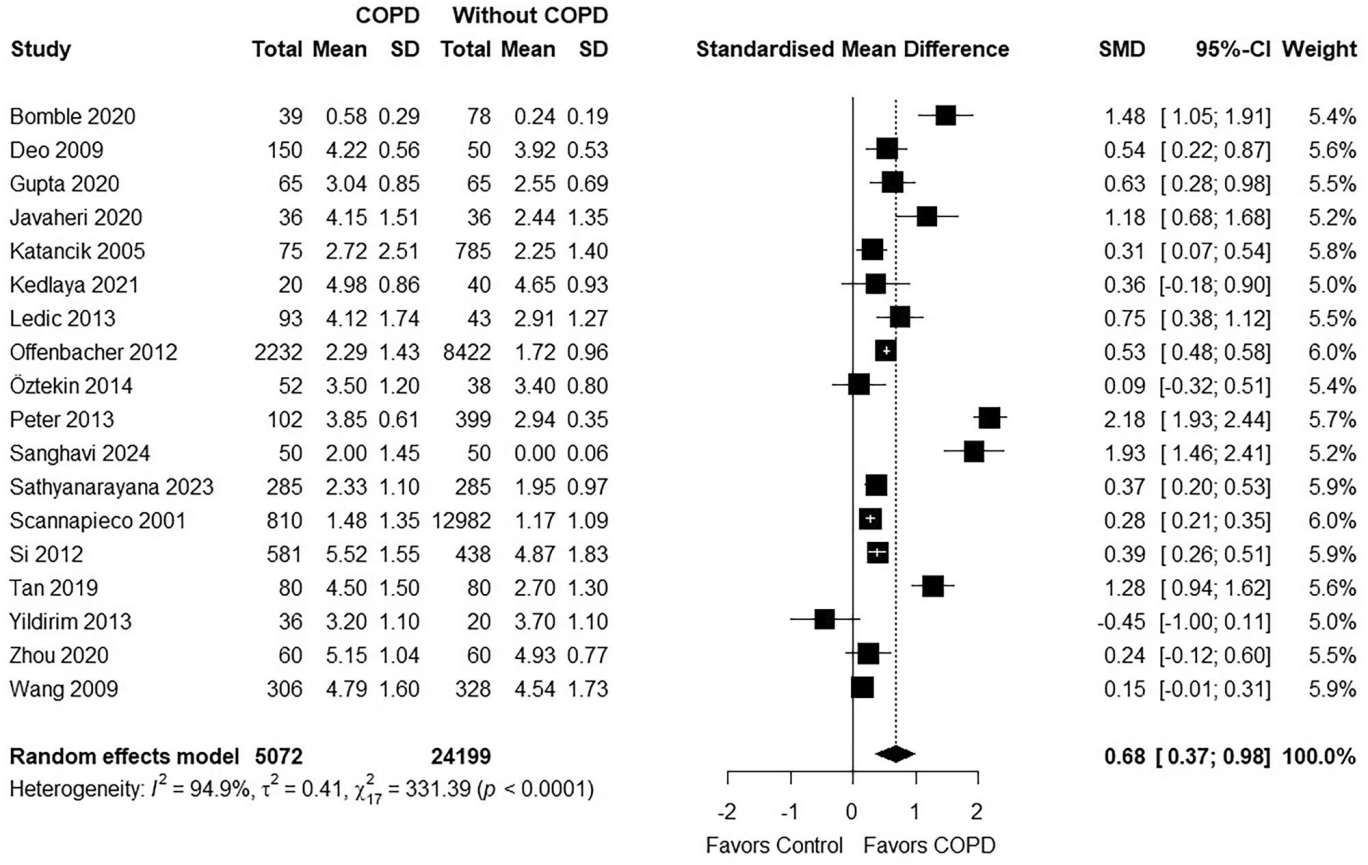
Forest plot for CAL.

Twenty-one studies [22, 25–27, 29, 32, 33, 35, 36, 38, 40, 47, 50, 52, 56–62] reported mean CAL measurements in patients with COPD, out of which two studies [36, 61] did not have a control group. One study by Sepolia et al. [59] provided huge SMD that is clinically implausible and was not included in the meta-analysis for generating pooled estimates. The pooled estimates (0.68 [0.37;0.98]) showed that the COPD group significantly showed higher mean CAL measurements compared to the group without COPD (Fig. 4). Leave-one-out sensitivity analysis further supported this finding, consistently showing significantly greater mean CAL in COPD patients when each study was omitted one at a time. (Supplementary File 11).

A Trim-and-Fill analysis was conducted to assess potential publication bias for this outcome. (Supplementary File 12) The method imputed two studies, and after adjustment, the recalculated pooled effect size was 0.49 (95% CI: 0.12; 0.86). After trim-and-fill adjustment, the pooled effect size was reduced, but remained statistically significant, suggesting that the finding is robust, although small-study effects, including possible publication bias, cannot be ruled out.

### Mean PD

**Fig. 5:**
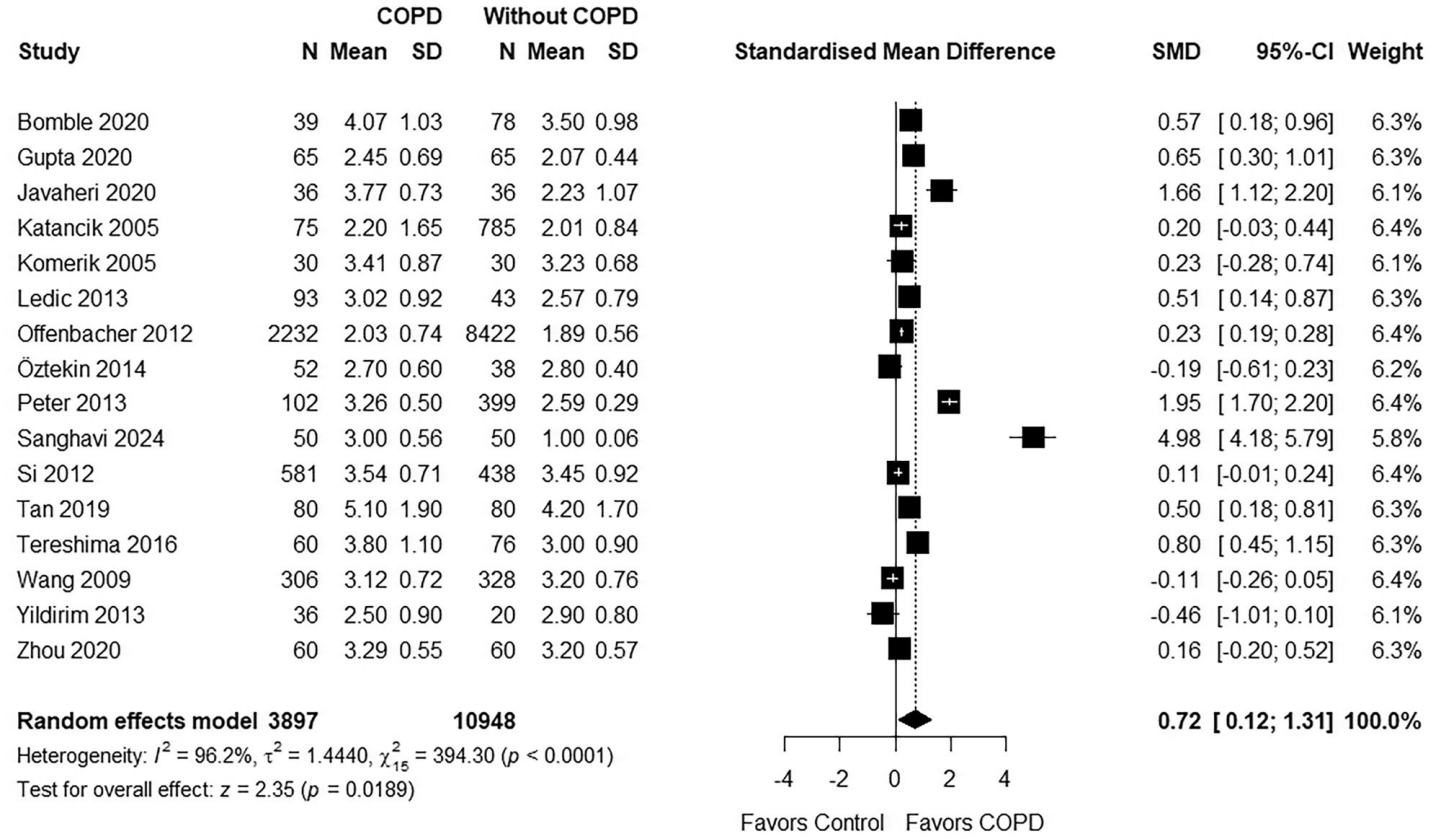
Forest plot for PD.

The mean pocket depth in COPD patients was reported in 19 studies [24, 25, 27, 29, 32, 33, 35, 36, 38, 40, 43, 47, 50, 52, 56, 57, 59, 61, 62]. Two studies did not have a control group [36, 61]. Sepolia et al. [59] provided huge SMD that is clinically implausible and so was not included in the meta-analysis. The pooled estimates showed that the participants in the COPD group has a significantly higher mean PD compared to the control group. (0.72 [0.12;1.31]) (Fig. 5) Leave-one-out sensitivity analysis (Supplementary File 13) further supported this finding, consistently showing significantly greater mean PD in COPD patients when each study was omitted one at a time.

A Trim-and-Fill analysis was conducted to assess potential publication bias for this outcome (Supplementary File 14). The Trim-and-Fill method imputed three studies. After adjusting for these missing studies, the recalculated pooled effect size was 0.24 (95% CI: −0.54; 1.01). The confidence interval crosses 0, indicating that the statistical significance of this association is sensitive to funnel plot asymmetry and potential small-study effects, including possible publication bias.

### CAL and PD greater than 3 mm

The number of patients with CAL and PD greater than 3 mm was reported in 14 studies [22, 23, 26, 30, 33, 39, 41, 43, 47, 48, 51, 53, 54, 59]. The odds ration revealed greater odds of CAL and PD greater than 3 mm in patients with COPD. The pooled estimates are presented in Supplementary File 15 and 16. COPD patients also had significantly higher odds (1.80 [0.82; 2.78]) of having PD greater than 5 mm compared with patients without COPD (Supplementary File 17).

### GRADE assessment

The certainty of evidence for primary outcomes was assessed using the GRADE approach (Table 4). The evidence for the association between COPD and periodontitis, mean CAL, and mean PD was rated as “Low” to “Very Low” certainty due to serious ROB, inconsistency, imprecision, and suspected publication bias. These ratings indicate that the true effects may differ substantially from the estimated effects with the emergence of new studies.

**Table 4:** GRADE certainty assessment for primary outcomes.

No of studies	Study design	Certainty assessment (GRADE)	Effect (95% CI)	Certainty
Outcome: Association COPD-periodontitis				
6	Observational	Risk of bias: Serious Inconsistency: Very serious Indirectness: Not serious Imprecision: Serious Publication bias: Strongly Suspected	OR 1.13 (0.66–1.91) After trim and fill	**⨁◯◯◯** VERY LOW
Outcome: Mean CAL (mm)				
21	Observational	Risk of bias: Serious Inconsistency: Very serious Indirectness: Not serious Imprecision: Serious Publication bias: Suspected	SMD 0.49 (0.12–0.87) After trim and fill	**⨁⨁◯◯** LOW
Outcome: Mean PD (mm)				
19	Observational	Risk of bias: Serious Inconsistency: Very serious Indirectness: Not serious Imprecision: Serious Publication bias: Strongly suspected	SMD 0.24 (−0.54 to 1.01) After trim and fill	**⨁◯◯◯** VERY LOW
Outcome: Mean BOP (%)				
4	Observational	Risk of bias: Serious Inconsistency: Very serious Indirectness: Not serious Imprecision: Not serious Publication bias: Undetected	SMD 1.49 (0.19–2.79)	**⨁⨁◯◯** LOW

Risk of bias: Downgraded for all outcomes due to inherent limitations of observational studies (residual confounding, selection bias) despite mostly satisfactory study quality.

Inconsistency: Downgraded for all outcomes due to substantial statistical heterogeneity (I² > 90%), with widely varying effect estimates across studies.

Imprecision: Downgraded for PD due to confidence intervals crossing the null.

Publication bias: Downgraded for outcomes with ≥10 studies based on trim-and-fill analysis suggesting missing studies would change significance. Not assessed for BOP (only 4 studies).

GRADE certainty levels:

VERY LOW (⨁◯◯◯): True effect is likely substantially different from estimate

LOW (⨁⨁◯◯): True effect may be substantially different from estimate

## Discussion

This meta-analysis indicates that periodontitis may be associated with COPD, as reflected by the higher prevalence of periodontal disease in individuals with COPD. Out of the 41 studies included, the pooled prevalence of periodontitis in COPD group was 35%, with moderate and severe periodontitis affecting 25% and 26% of individuals, respectively. This aligns with previous research suggesting that COPD patients are at an elevated risk of periodontitis due to common pathophysiological mechanisms, such as chronic inflammation and immune dysregulation [64]. The association between COPD and periodontitis was further supported by the analysis of various clinical parameters, including BOP, CAL, and PD, all of which were higher in COPD patients compared to controls. These results suggest a possible relationship between COPD and periodontitis, highlighting the importance of integrated care for both respiratory and oral health in this population.

Confounding factors may play a significant role in the observed association between periodontitis and COPD [18]. Key confounders include smoking status, age, socioeconomic status, systemic conditions such as diabetes and cardiovascular disease, medication use (e.g., corticosteroids and bronchodilators), and oral hygiene practices. Smoking, in particular, is a major shared risk factor that contributes to both conditions by promoting chronic inflammation, immune dysregulation, and microbiome alterations [57]. A meta-analysis on observational studies by Yang et al. [18] showed that the pooled analysis of 18 studies suggested that periodontitis was weakly associated with the risk of COPD (OR: 1.20, 95% CI 1.09–1.32). However, in stratified and subgroup analyses of 10 studies, with strict adjustment for smoking, periodontitis was no longer related to the risk of COPD (adjusting for smoking intensity: OR: 1.14, 95% CI 0.86–1.51) Moreover, periodontal disease did not increase the risk of COPD-related exacerbation or mortality (OR: 1.18, 95% CI 0.71–1.97) in the pooled result of four studies [18]. In our study, stratified analysis revealed a pooled periodontitis prevalence of 44% among COPD patients with more than 50% smokers, compared to a markedly lower prevalence of 19% in those with less than 50% smokers. Several included studies in this analysis reported a high proportion of smokers among COPD patients, highlighting the widespread prevalence of smoking in this population and the challenge of disentangling its specific impact on periodontal health. This difference underscores the potential amplifying effect of smoking on periodontal disease in individuals with COPD. A key limitation of our analysis is the limited number of studies that adequately adjusted for other confounding variables such as comorbidities, medication use, and socioeconomic status. Nevertheless, the consistent significance of periodontal parameters across studies suggests a possible independent association between periodontitis and COPD, beyond the influence of smoking alone.

For non-smokers with COPD, the risk factors are more varied and include exposure to biomass, occupational hazards, passive smoking, as well as a history of asthma, tuberculosis, or respiratory infections during childhood [65]. The effect of residential radon on COPD risk remains unclear [65]. Notably, previous respiratory diseases of any type represent the highest risk, with a magnitude much higher than that observed for other factors [65]. However, data on the confounders influencing periodontal health of COPD patients who have never smoked is scarce. This highlights the need for further research to better understand the specific periodontal risks in this subgroup. The limited data available on non-smokers with COPD is a significant gap in the current literature, representing a key limitation of our analysis. Future studies with individual patient data and rigorous confounder adjustments will be necessary to establish causality and further validate these findings.

This meta-analysis has several limitations that should be considered. Considerable heterogeneity was observed across studies, likely due to evolving periodontal classification systems, varying diagnostic thresholds, and differences in measurement protocols, as reflected by high I² values. Only three parameters (BOP, CAL, and PD) were included, and the review was restricted to peer-reviewed publications indexed in major databases; grey literature was not searched, which may have increased the risk of publication bias. Crucially, the overall certainty of evidence was graded as low to very low. While CAL showed a significant association, findings related to PD should be interpreted with particular caution, as the statistical significance of the pooled PD estimates was sensitive to trim-and-fill adjustment and could be influenced by small-study effects or publication bias. While the consistent trend across multiple parameters suggests a potential link, these methodological constraints preclude definitive causal inferences. The limited data on non-smokers with COPD represents a significant gap, making it difficult to disentangle the effects of smoking from a potential independent association. Therefore, these results should be interpreted as indicative of an association that requires further validation.

To conclude, this comprehensive meta-analysis provides pooled evidence that COPD may be associated with worse clinical periodontal status. The strong modifying effect of smoking status on periodontitis prevalence underscores its role as a major shared risk factor. While our rigorous sensitivity analyses and assessment highlight the need for cautious interpretation, the consistency of the association across multiple parameters suggests a clinically relevant link between pulmonary and oral health. Future research should prioritize longitudinal studies with standardized periodontal case definitions and detailed adjustment for smoking and other shared risk factors to clarify causality and explore the impact of periodontal therapy on COPD progression and exacerbations.

## Supplementary information

Supplemental File 1- Search strategy

Supplemental File 2- NOS categories

Supplemental File 3- Stratified for smoking greater than 50

Supplemental File 4- Stratified for smoking less than 50

Supplemental File 5- Perio prevalence based on study types

Supplemental File 6- Leave one out Periodontitis Prev

Supplemental File 7- Funnel plot periodontitis prevalence

Supplemental File 8- Leave one out periodontitis OR

Supplemental File 9- Funnel plot Periodontitis OR

Supplemental File 10- Forest plot BOP

Supplemental File 11- Leave one out CAL

Supplemental File 12- Funnel plot CAL

Supplemental File 13- Leave one out PD

Supplemental File 14- Funnel plot for PD

Supplemental File 15- Forest plot for PD(3mm)

Supplemental File 16- Forest plot for CAL(3mm)

Supplemental File 17- Forest plot for PD(5mm)

## Data Availability

The datasets used and/or analysed during the current study are available from the corresponding author on reasonable request.
