# Supplementary material for: Linking lungs and gums: a meta-analysis of periodontitis prevalence and severity in chronic obstructive pulmonary disease
Source: BDJ Open. 2026 Feb 9;12:16. doi: 10.1038/s41405-026-00403-6 (PMC12887045; doi:10.1038/s41405-026-00403-6)
Supplement: Supplementary file 1 — Supplemental File 1- Search strategy [file 41405_2026_403_MOESM1_ESM.pdf]

Supplemental File 1- Search strategy:

EMBASE

('chronic obstructive lung disease':ab,kw,ti OR 'copd':ab,kw,ti OR 'obstructive lung disease':ab,kw,ti OR 'asthma':ab,ti,kw OR 'respiratory tract disease':ab,kw,ti OR 'wheezing':ab,kw,ti) AND ('periodontal disease':kw,ab,ti OR 'tooth loss':ab,kw,ti OR 'chronic periodontitis':ab,kw,ti OR 'periodontitis':kw,ab,ti OR 'aggressive periodontitis'/exp OR 'aggressive periodontitis')

PUBMED

(("Chronic Obstructive Pulmonary Disease"[Title/Abstract] OR "COPD"[Title/Abstract] OR "Obstructive Lung Disease"[Title/Abstract] OR "Asthma"[Title/Abstract] OR "Respiratory Tract Disease"[Title/Abstract] OR "Wheezing"[Title/Abstract] OR "Pulmonary Disease, Chronic Obstructive"[MeSH] OR "Asthma"[MeSH] OR "Respiratory Tract Diseases"[MeSH])) AND (("Periodontal Disease"[Title/Abstract] OR "Tooth Loss"[Title/Abstract] OR "Chronic Periodontitis"[Title/Abstract] OR "Periodontitis"[Title/Abstract] OR "Aggressive Periodontitis"[Title/Abstract] OR "Periodontal Diseases"[MeSH] OR "Tooth Loss"[MeSH] OR "Periodontitis"[MeSH]))

SCOPUS

(TITLE-ABS-KEY ("chronic obstructive pulmonary disease") OR TITLE-ABS-KEY ("COPD") OR TITLE-ABS-KEY ("obstructive lung disease") OR TITLE-ABS-KEY ("asthma") OR TITLE-ABS-KEY ("respiratory tract disease") OR TITLE-ABS-KEY ("wheezing")) AND (TITLE-ABS-KEY ("periodontal disease") OR TITLE-ABS-KEY ("tooth loss") OR TITLE-ABS-KEY ("chronic periodontitis") OR TITLE-ABS-KEY ("periodontitis") OR TITLE-ABS-KEY ("aggressive periodontitis"))

WOS

TS=("chronic obstructive pulmonary disease" OR "COPD" OR "obstructive lung disease" OR "asthma" OR "respiratory tract disease" OR "wheezing") AND TS=("periodontal disease" OR "tooth loss" OR "chronic periodontitis" OR "periodontitis" OR "aggressive periodontitis")
