# Supplementary material for: Linking lungs and gums: a meta-analysis of periodontitis prevalence and severity in chronic obstructive pulmonary disease
Source: BDJ Open. 2026 Feb 9;12:16. doi: 10.1038/s41405-026-00403-6 (PMC12887045; doi:10.1038/s41405-026-00403-6)
Supplement: Supplementary file 2 — Supplemental File 2- NOS categories [file 41405_2026_403_MOESM2_ESM.pdf]

NEWCASTLE - OTTAWA QUALITY ASSESSMENT SCALE

Note: A study was awarded a maximum of one points for each numbered item within the Selection and Outcome categories. A maximum of two points can be given for Comparability.

Rating Scale: Good (5-7), Satisfactory (4-3), Unsatisfactory (>2)

Selection (Maximum 4 points)

1) *Representativeness of the exposed cohort*

a) Recruitment of COPD patients in the community (+1)

b) Recruitment of COPD patients in from Hospital or clinics only

c) No description of the derivation of the cohort

2) *Sample size*

a) Justified and satisfactory (+1)

b) Not justified

3) *Ascertainment of exposure*

a) Patient record (e.g. surgical records) (+1)

b) Structured interview (+1)

c) Written self-report

d) No description

4) *Sampling method*

a) Appropriate sampling. (+1)

b) The response rate is unsatisfactory, or the comparability between respondents and non-respondents is unsatisfactory.

c) No description of the response rate or the characteristics of the responders and the non-responders.

Comparability (Maximum 2 points)

1) Included control group without COPD irrespective of outcome

a) Yes (+1)

b) No

Outcome (Maximum 3 stars)

1) Assessment of outcome

a) Independent blind assessment (+1)

b) Record linkage (+1)

c) Self-report

d) No description

2) Was follow-up long enough for outcomes to occur

a) Yes (+1)

b) No (0)

3) Adequacy of follow up

a) Complete follow up or adequate duration of exposure (+1)

b) Subjects lost to follow up unlikely to introduce bias (+1)

c) Follow up rate < 90 % and no description of those lost

d) No statement
