# Supplementary figures and images for: Linking lungs and gums: a meta-analysis of periodontitis prevalence and severity in chronic obstructive pulmonary disease
Source: BDJ Open. 2026 Feb 9;12:16. doi: 10.1038/s41405-026-00403-6 (PMC12887045; doi:10.1038/s41405-026-00403-6)

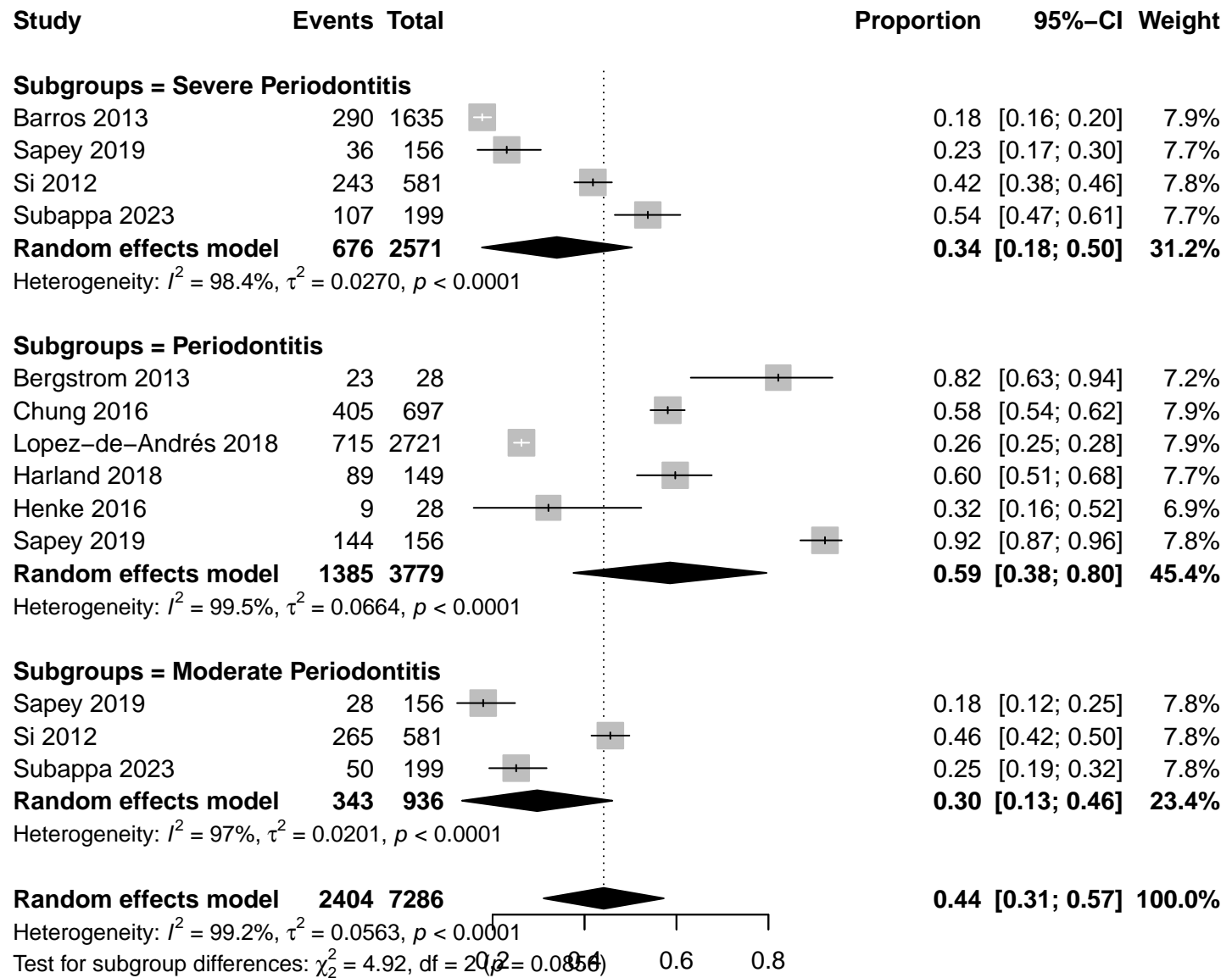

Supplement: Supplementary file 3 — Supplemental File 3- Stratified for smoking greater than 50 [file 41405_2026_403_MOESM3_ESM.pdf]

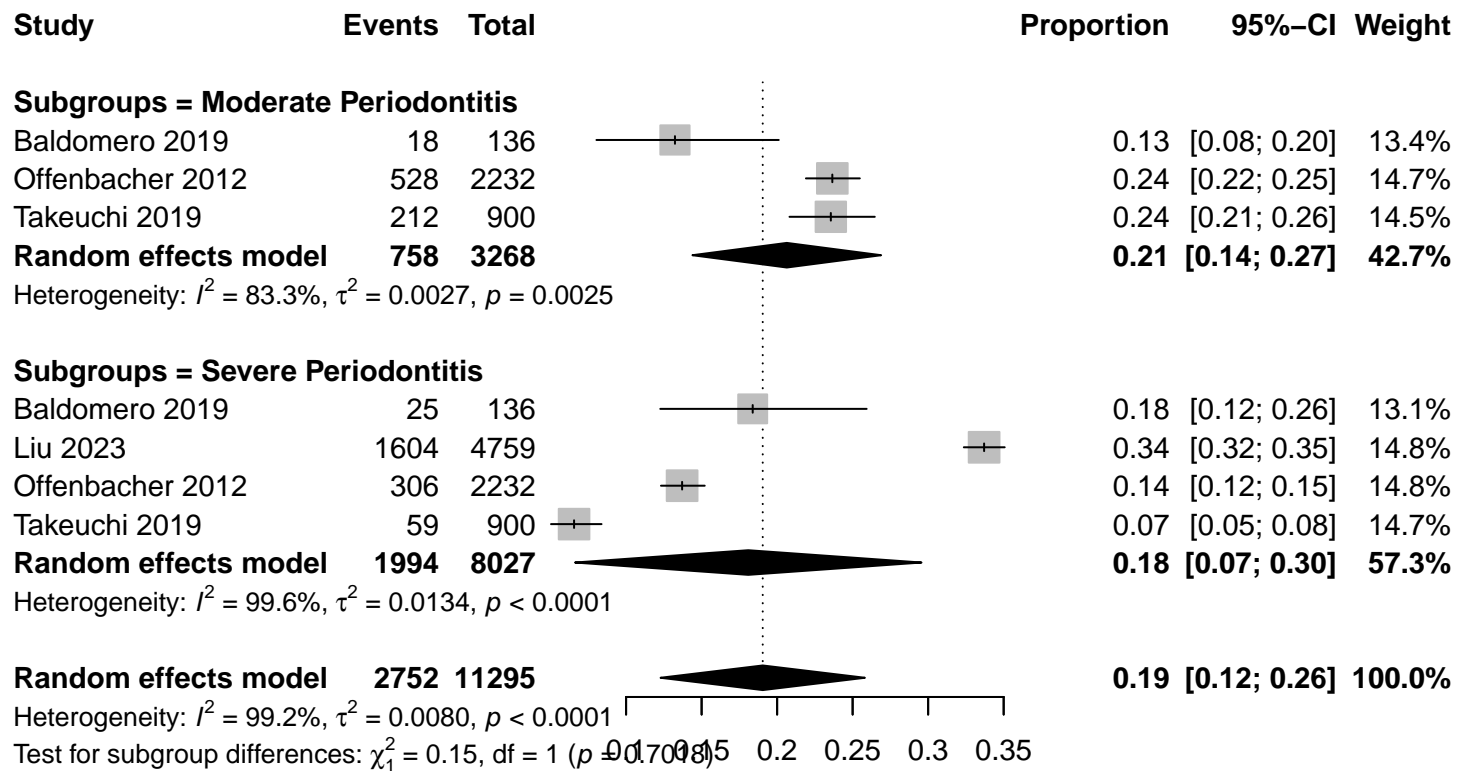

Supplement: Supplementary file 4 — Supplemental File 4- Stratified for smoking less than 50 [file 41405_2026_403_MOESM4_ESM.pdf]

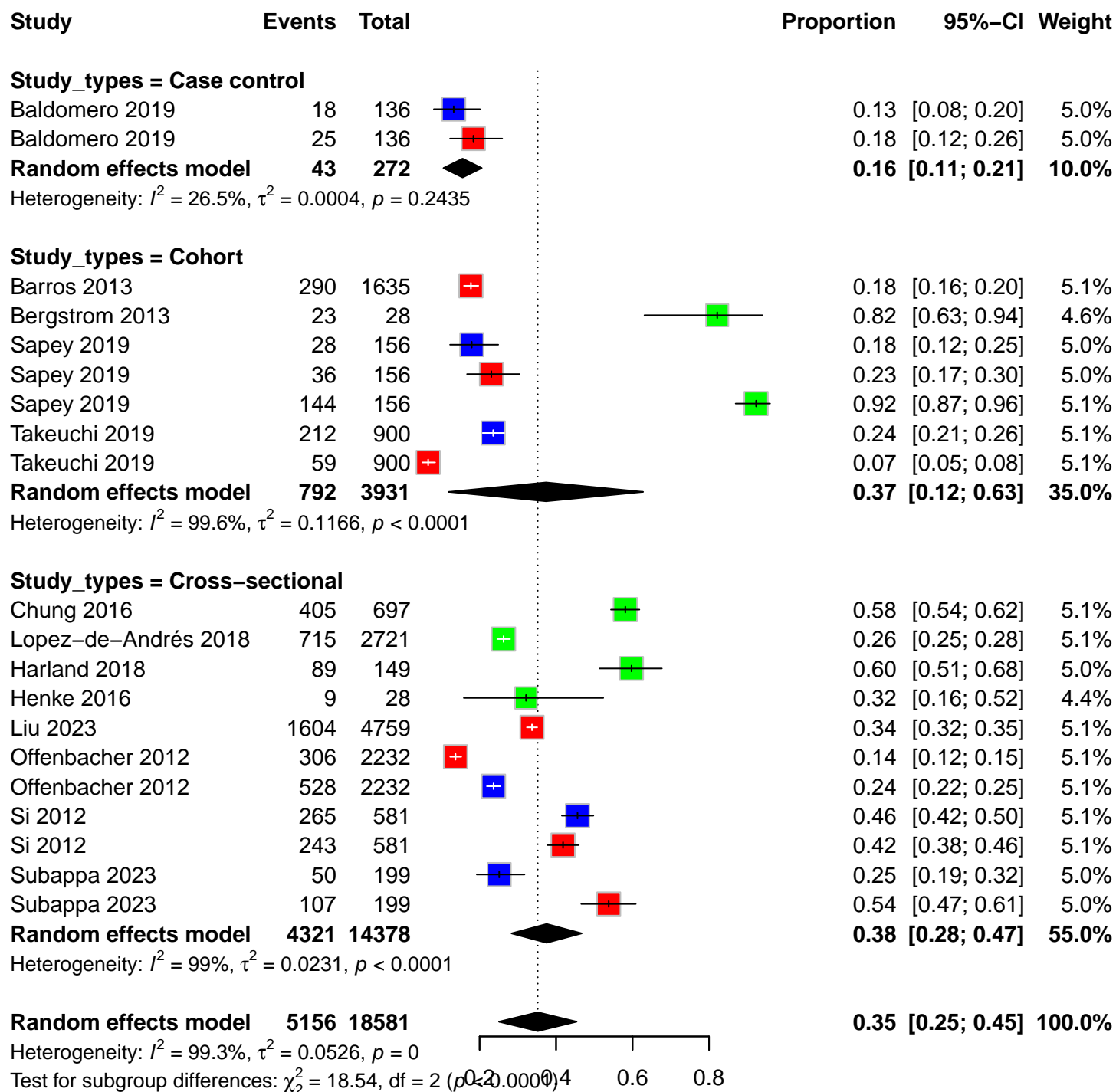

Supplement: Supplementary file 5 — Supplemental File 5- Perio prevalence based on study types [file 41405_2026_403_MOESM5_ESM.pdf]

Leave-One-Out Analysis for Prevalence

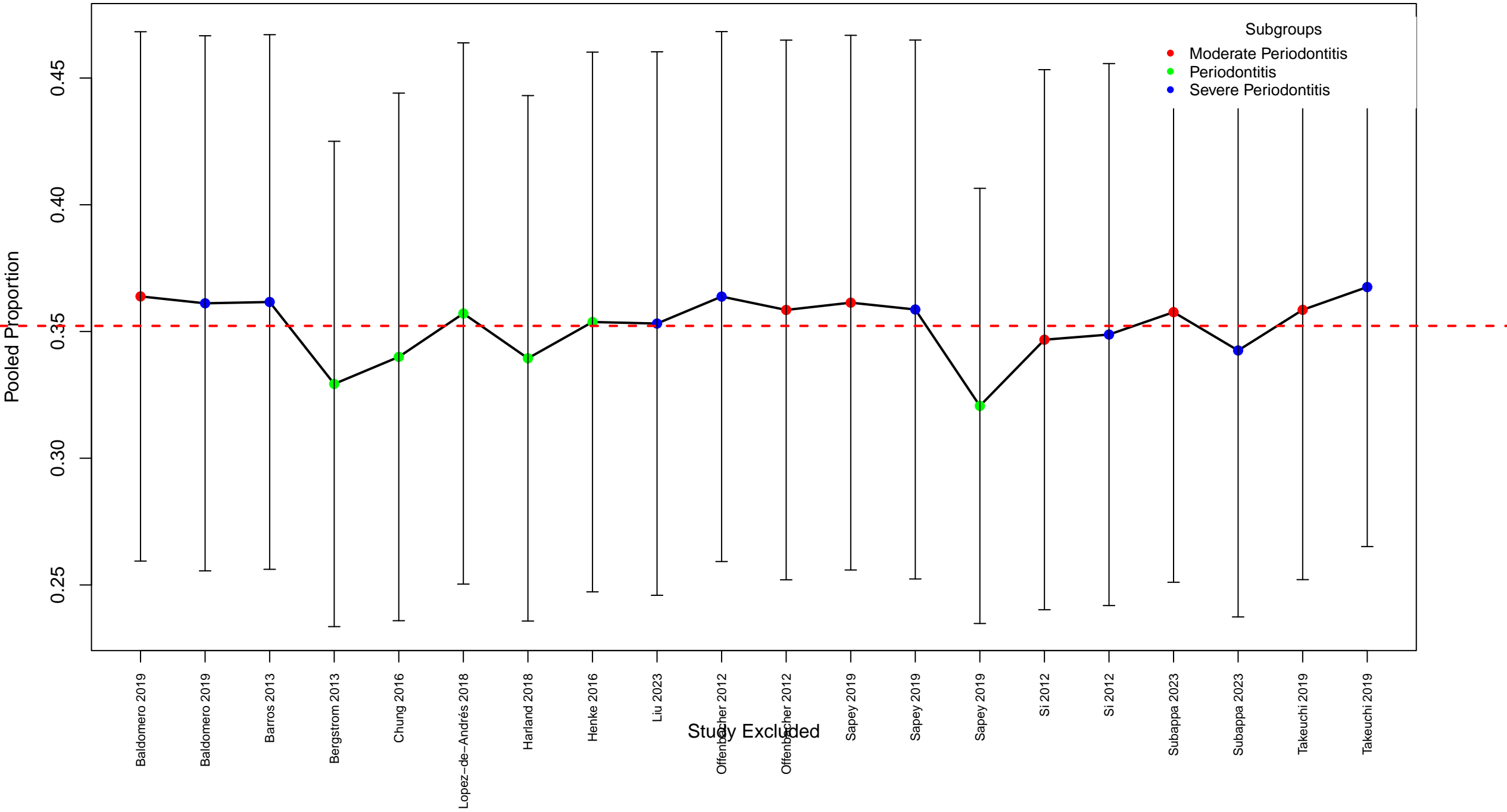

Supplement: Supplementary file 6 — Supplemental File 6- Leave one out Periodontitis Prev [file 41405_2026_403_MOESM6_ESM.pdf]

Funnel Plot with Trim and Fill Adjustment for Periodontitis Prevalence

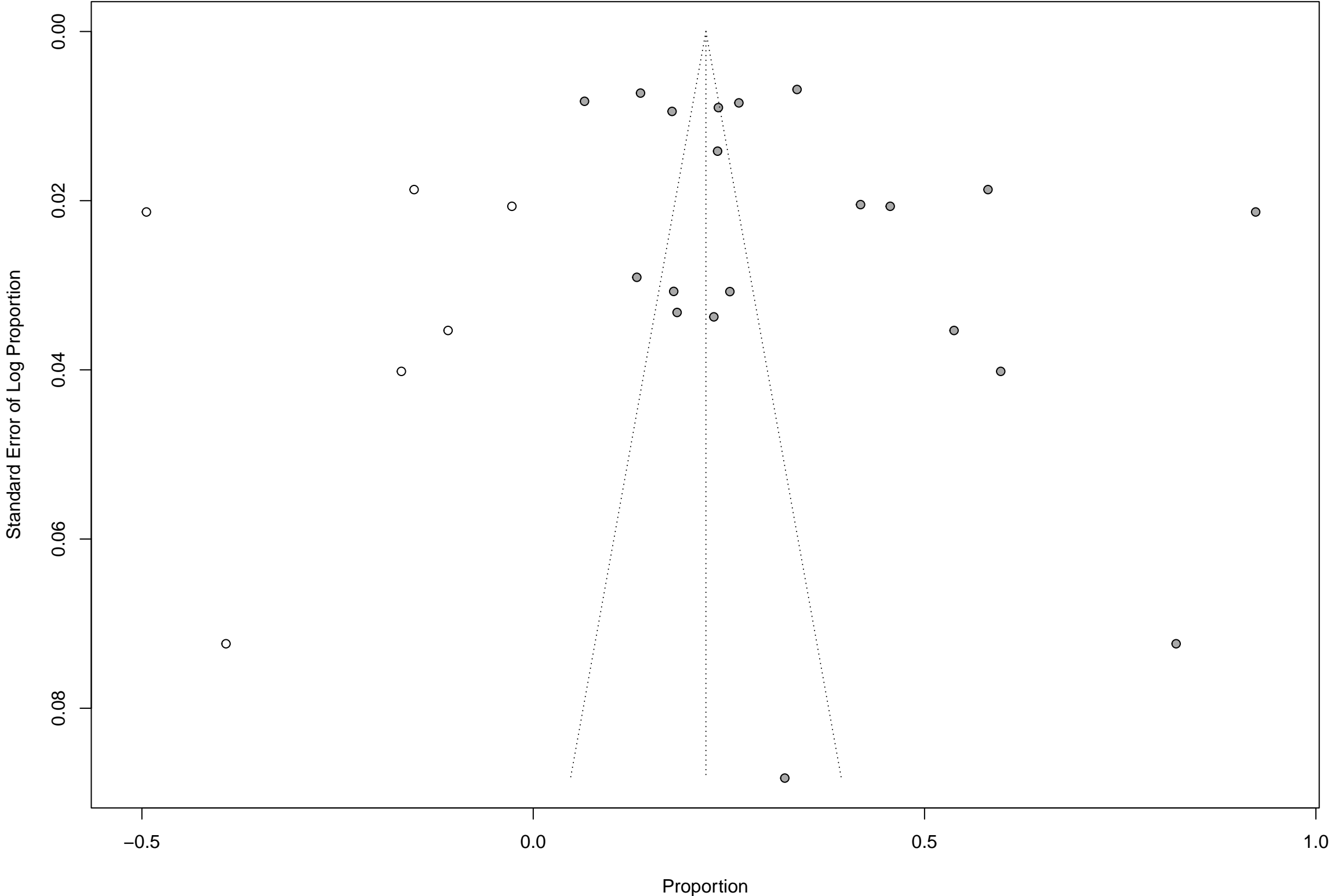

Supplement: Supplementary file 7 — Supplemental File 7- Funnel plot periodontitis prevalence [file 41405_2026_403_MOESM7_ESM.pdf]

Leave-One-Out Analysis for Periodontitis (OR)

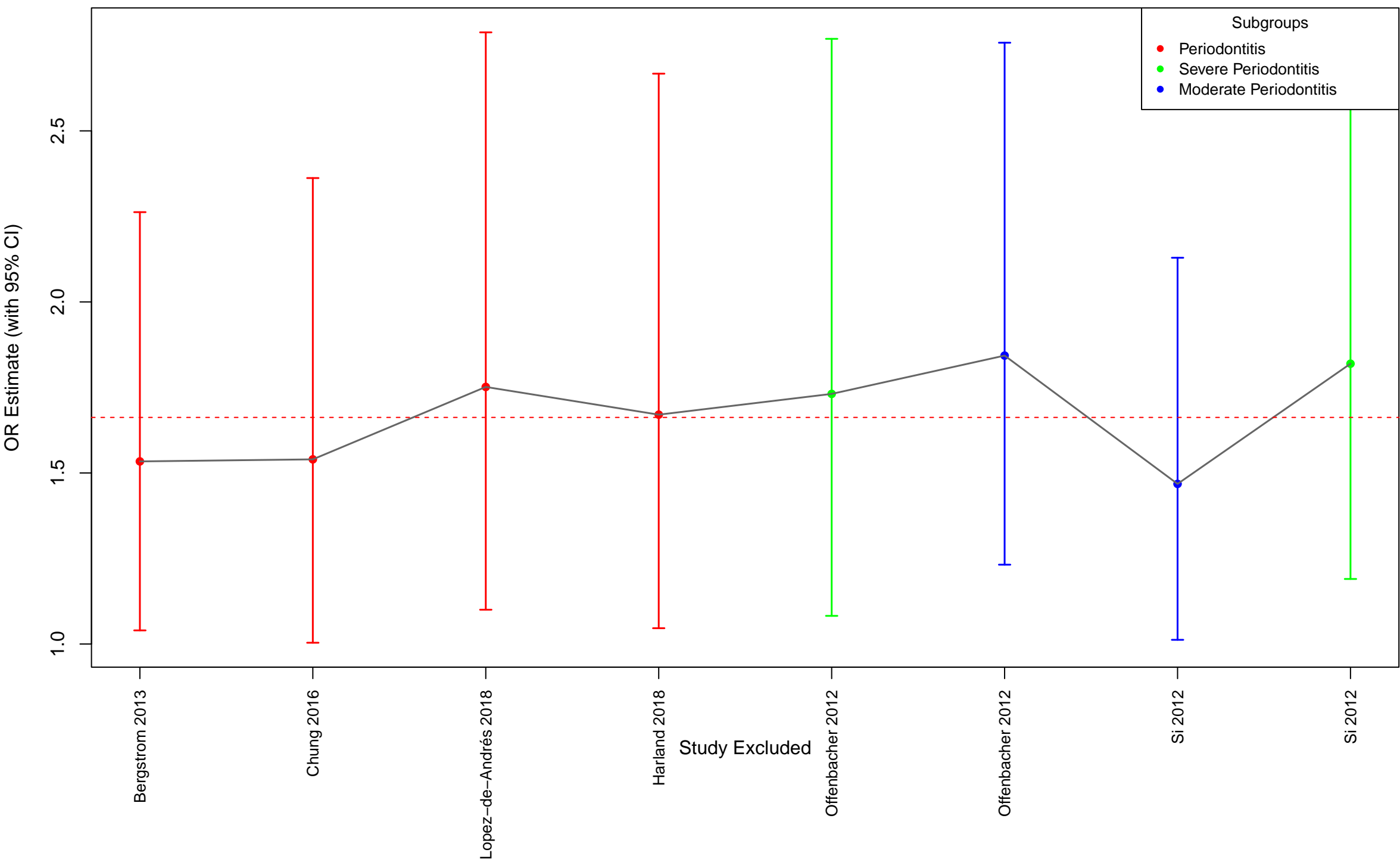

Supplement: Supplementary file 8 — Supplemental File 8- Leave one out periodontitis OR [file 41405_2026_403_MOESM8_ESM.pdf]

Funnel Plot with Trim and Fill Adjustment for Periodontitis OR

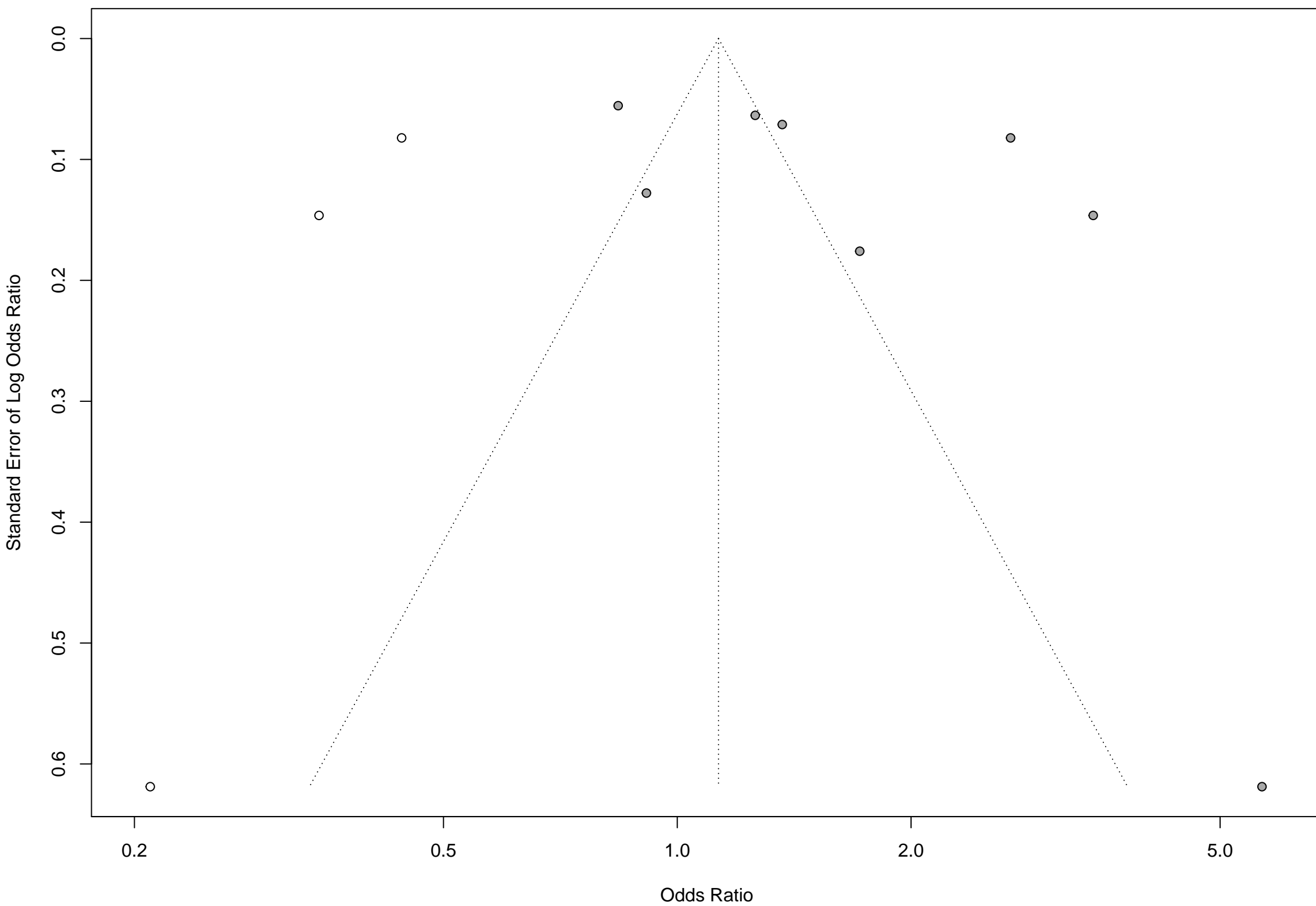

Supplement: Supplementary file 9 — Supplemental File 9- Funnel plot Periodontitis OR [file 41405_2026_403_MOESM9_ESM.pdf]

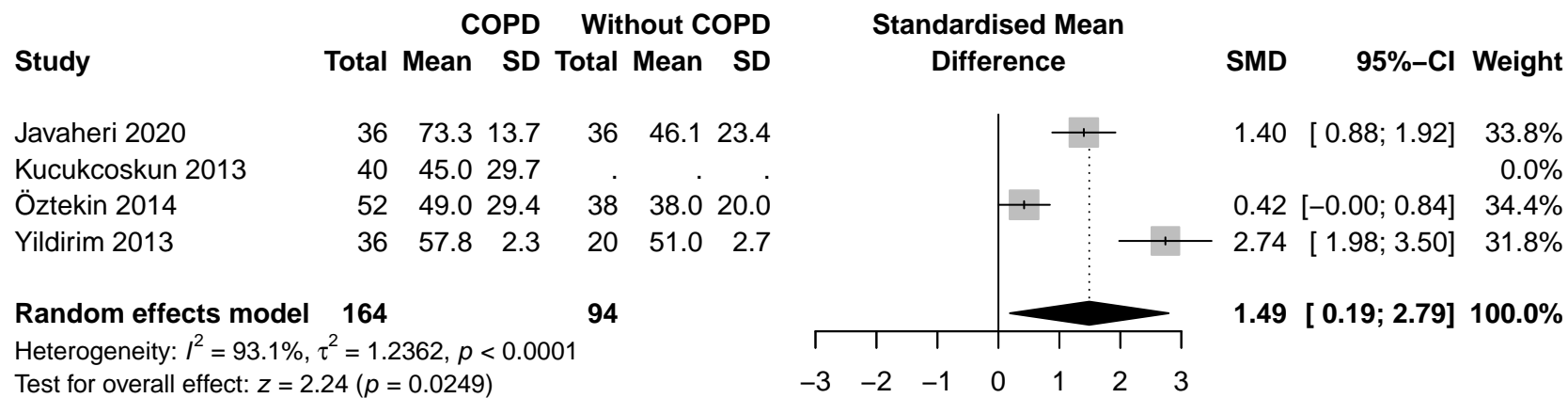

Supplement: Supplementary file 10 — Supplemental File 10- Forest plot BOP [file 41405_2026_403_MOESM10_ESM.pdf]

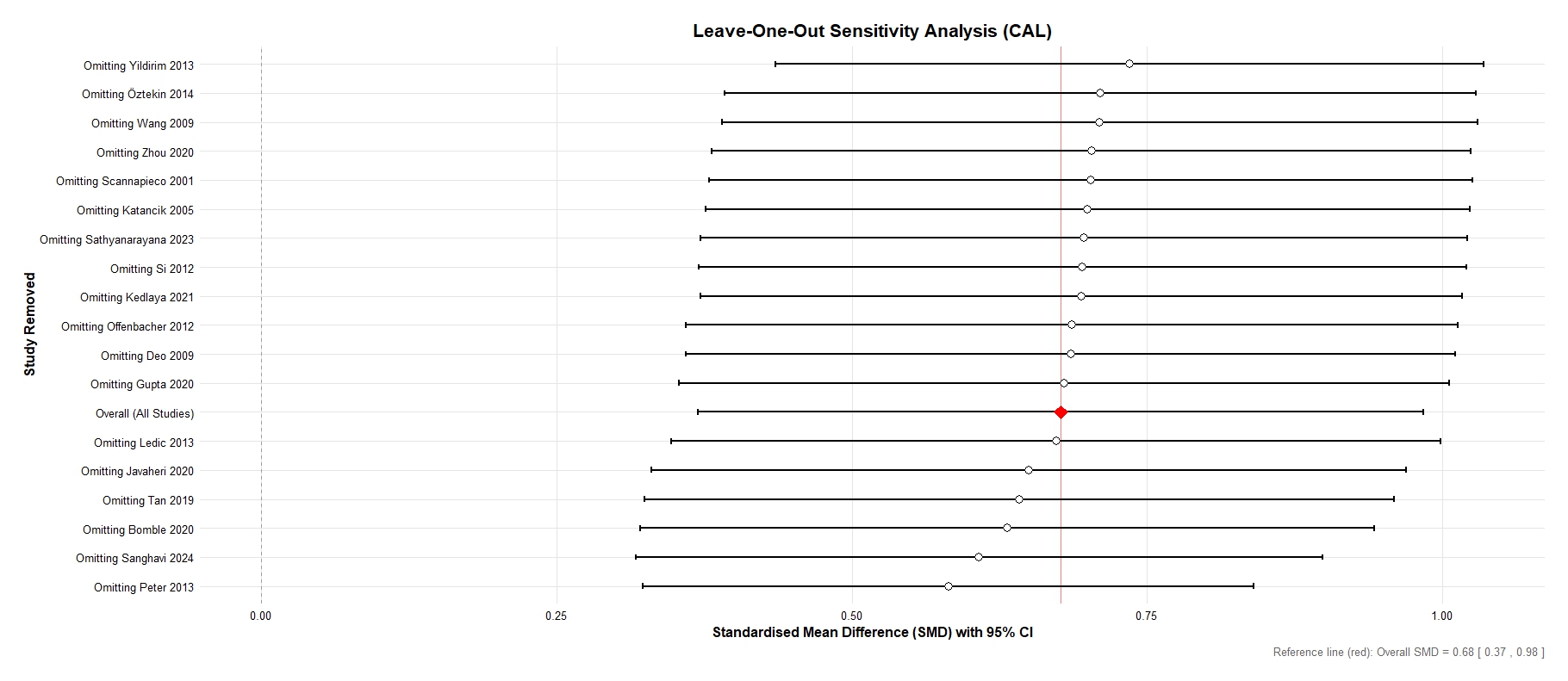

Supplement: Supplementary file 11 — Supplemental File 11- Leave one out CAL [file 41405_2026_403_MOESM11_ESM.jpg]

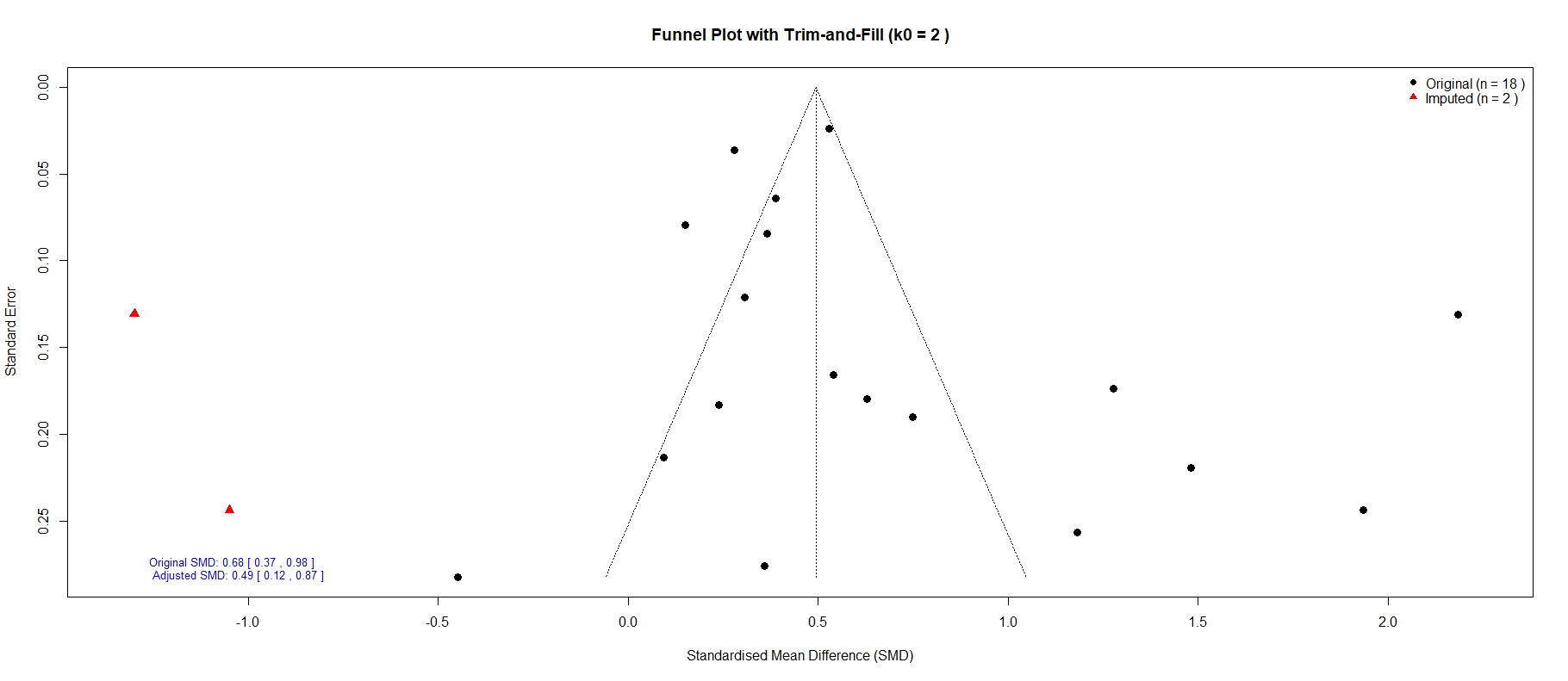

Supplement: Supplementary file 12 — Supplemental File 12- Funnel plot CAL [file 41405_2026_403_MOESM12_ESM.jpg]

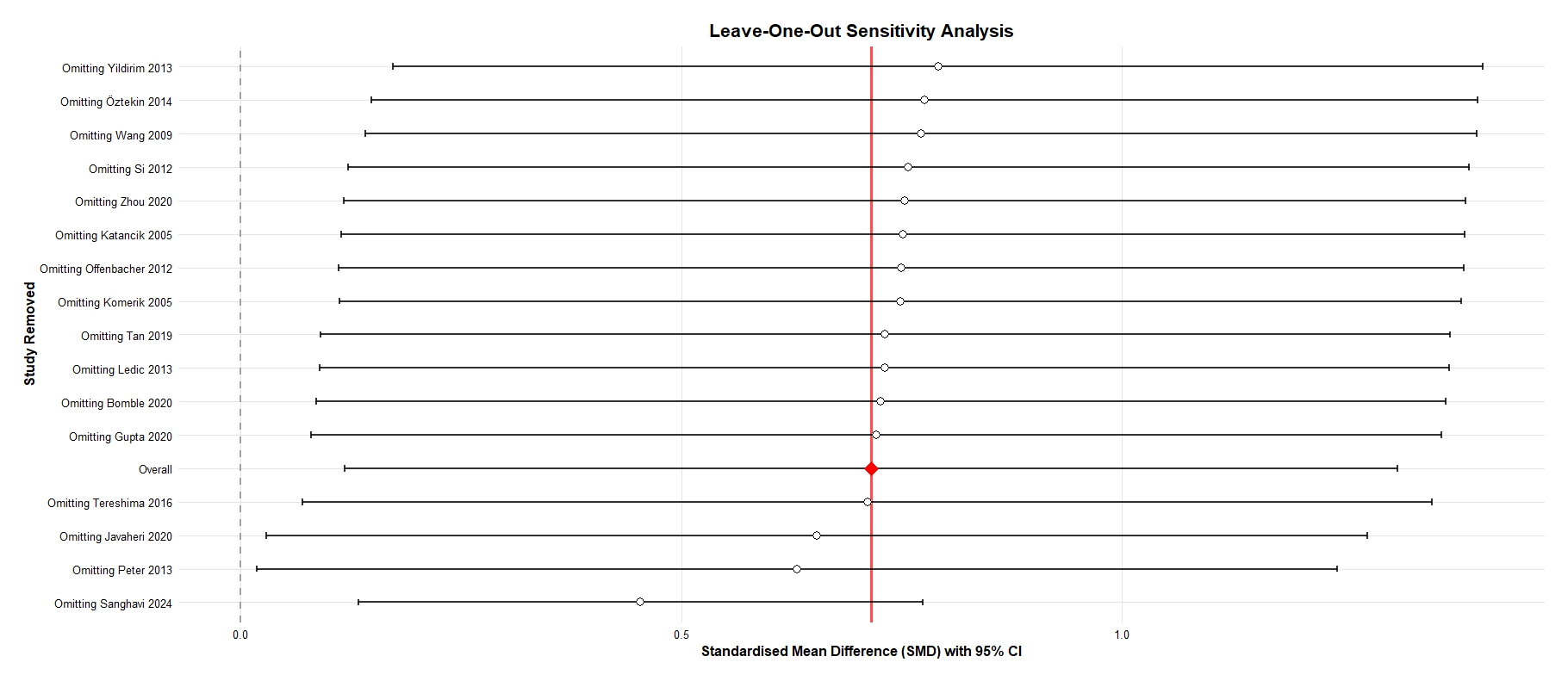

Supplement: Supplementary file 13 — Supplemental File 13- Leave one out PD [file 41405_2026_403_MOESM13_ESM.jpg]

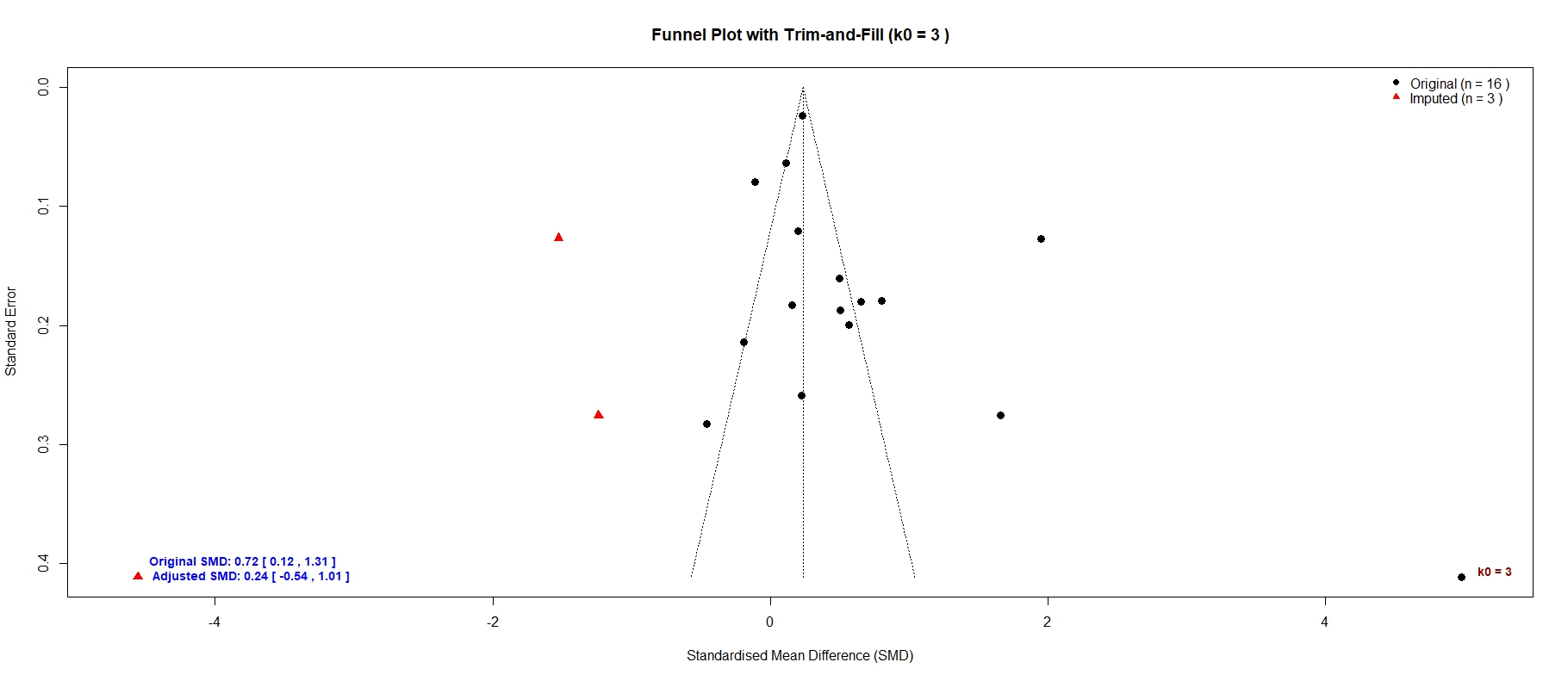

Supplement: Supplementary file 14 — Supplemental File 14- Funnel plot for PD [file 41405_2026_403_MOESM14_ESM.jpg]

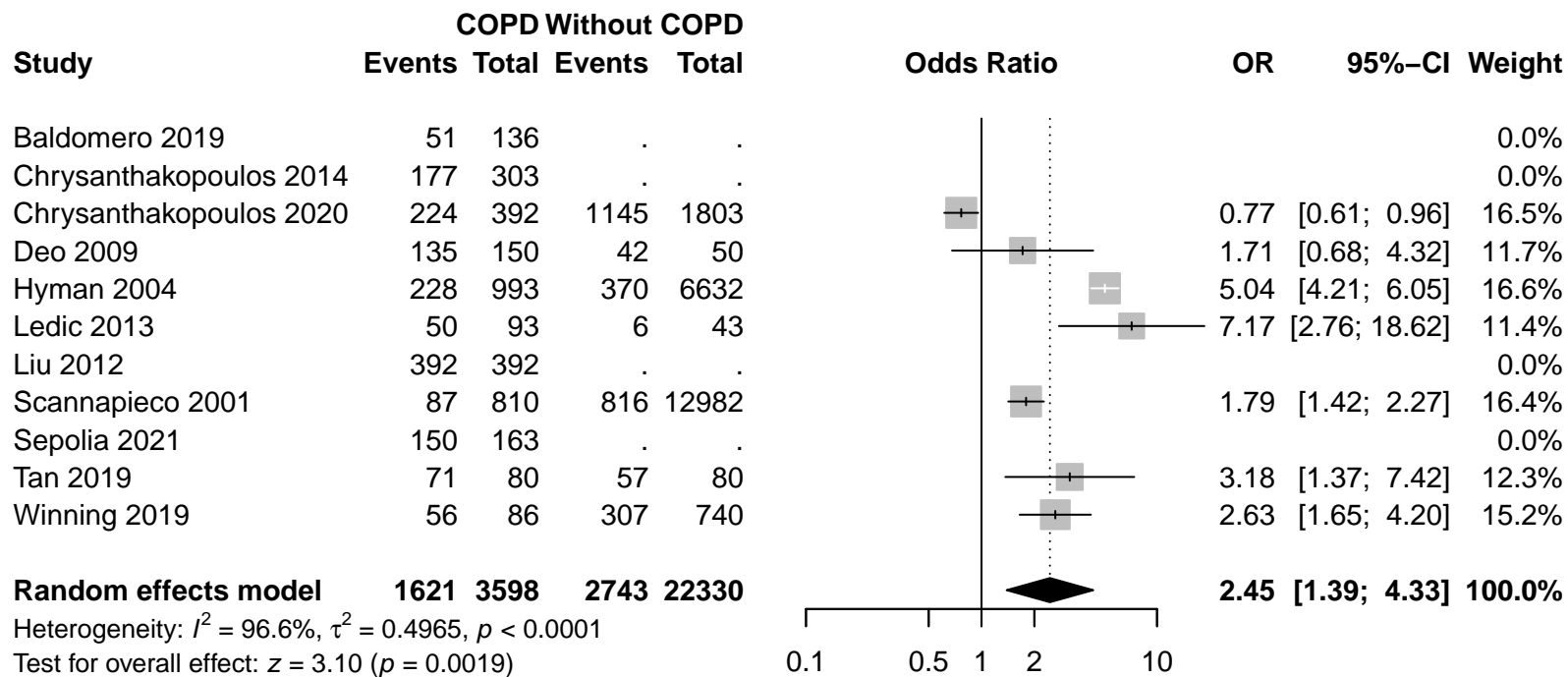

Supplement: Supplementary file 16 — Supplemental File 16- Forest plot for CAL(3mm) [file 41405_2026_403_MOESM16_ESM.pdf]
