# Supplementary material for: Linking lungs and gums: a meta-analysis of periodontitis prevalence and severity in chronic obstructive pulmonary disease
Source: BDJ Open. 2026 Feb 9;12:16. doi: 10.1038/s41405-026-00403-6 (PMC12887045; doi:10.1038/s41405-026-00403-6)
Supplement: Supplementary file 15 — Supplemental File 15- Forest plot for PD(3mm) [file 41405_2026_403_MOESM15_ESM.pdf]

Study	COPD Events	COPD Total	Without COPD Events	Without COPD Total	OR	95%−CI	Weight
Baldomero 2019	29	136	.	.			0.0%
Chrysanthakopoulos 2014	179	302	.	.			0.0%
Chrysanthakopoulos 2020	392	392	1803	1803			0.0%
Chung 2016	404	697	1764	5181	2.67	[2.27; 3.14]	30.9%
Hyman 2004	35	993	141	6632	1.68	[1.15; 2.45]	18.8%
Jung 2020	948	1134	4898	6585	1.76	[1.49; 2.07]	30.5%
Liu 2012	191	392	.	.			0.0%
Tan 2019	52	80	34	80	2.51	[1.33; 4.76]	9.9%
Winning 2019	13	86	69	740	1.73	[0.91; 3.28]	9.9%
**Random effects model**	**2243**	**4212**	**8709**	**21021**	**2.05**	**[1.62; 2.60]**	**100.0%**

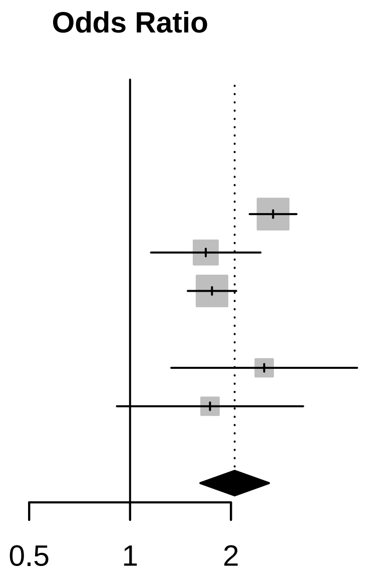

Heterogeneity: $I^2 = 73.2\%$, $\tau^2 = 0.0403$, $p = 0.0048$

Test for overall effect: $z = 5.96$ ($p < 0.0001$)
