# Supplementary material for: Linking lungs and gums: a meta-analysis of periodontitis prevalence and severity in chronic obstructive pulmonary disease
Source: BDJ Open. 2026 Feb 9;12:16. doi: 10.1038/s41405-026-00403-6 (PMC12887045; doi:10.1038/s41405-026-00403-6)

Study	COPD Events	COPD Total	Without COPD Events	Without COPD Total	OR	95%−CI	Weight
Baldomero 2019	7	136	.	.			0.0%
Chrysanthakopoulos 2014	179	302	.	.			0.0%
Chrysanthakopoulos 2020	229	393	1153	1804	0.79	[0.63; 0.98]	28.6%
Chung 2016	130	697	497	5181	2.16	[1.75; 2.67]	28.8%
Tan 2019	52	80	34	80	2.51	[1.33; 4.76]	21.3%
Winning 2019	13	86	69	740	1.73	[0.91; 3.28]	21.3%
**Random effects model**	**610**	**1694**	**1753**	**7805**	**1.60**	**[0.92; 2.76]**	**100.0%**

Heterogeneity: $I^2 = 93.4\%$, $\tau^2 = 0.2593$, $p < 0.0001$

Test for overall effect: $z = 1.67$ ($p = 0.0944$)

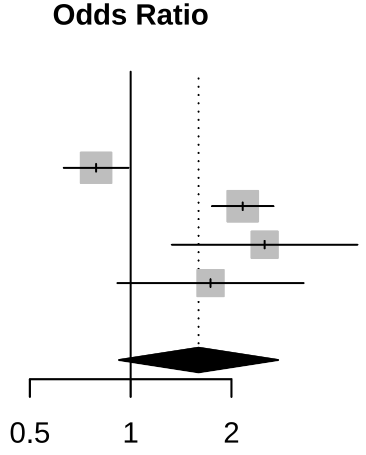

Supplement: Supplementary file 17 — Supplemental File 17- Forest plot for PD(5mm) [file 41405_2026_403_MOESM17_ESM.pdf]
